# Small molecule inhibitors for cancer immunotherapy and associated biomarkers – the current status

**DOI:** 10.3389/fimmu.2023.1297175

**Published:** 2023-10-31

**Authors:** Lisa Schlicher, Luke G. Green, Andrea Romagnani, Florian Renner

**Affiliations:** ^1^ Cancer Cell Targeted Therapy, Roche Pharma Research and Early Development, Roche Innovation Center Basel, F. Hoffmann-La Roche AG, Basel, Switzerland; ^2^ Therapeutic Modalities, Roche Pharma Research and Early Development, Roche Innovation Center Basel, F. Hoffmann-La Roche AG, Basel, Switzerland

**Keywords:** cancer immunotherapy, small molecule inhibitors, immuno-oncology, tumor microenvironment, adenosine, cGAS/STING, T-cell receptor signaling, biomarker

## Abstract

Following the success of cancer immunotherapy using large molecules against immune checkpoint inhibitors, the concept of using small molecules to interfere with intracellular negative regulators of anti-tumor immune responses has emerged in recent years. The main targets for small molecule drugs currently include enzymes of negative feedback loops in signaling pathways of immune cells and proteins that promote immunosuppressive signals within the tumor microenvironment. In the adaptive immune system, negative regulators of T cell receptor signaling (MAP4K1, DGKα/ζ, CBL-B, PTPN2, PTPN22, SHP1), co-receptor signaling (CBL-B) and cytokine signaling (PTPN2) have been preclinically validated as promising targets and initial clinical trials with small molecule inhibitors are underway. To enhance innate anti-tumor immune responses, inhibitory immunomodulation of cGAS/STING has been in the focus, and inhibitors of ENPP1 and TREX1 have reached the clinic. In addition, immunosuppressive signals via adenosine can be counteracted by CD39 and CD73 inhibition, while suppression via intratumoral immunosuppressive prostaglandin E can be targeted by EP2/EP4 antagonists. Here, we present the status of the most promising small molecule drug candidates for cancer immunotherapy, all residing relatively early in development, and the potential of relevant biomarkers.

## Introduction

Among cancer therapies, immune checkpoint inhibition using antibodies against programmed cell death protein 1 (PD-1), PD-1 ligand (PD-L1), or cytotoxic T-lymphocyte-associated protein 4 (CTLA-4) has led to impressive clinical responses in patients. However, not all patients benefit from immune checkpoint inhibitors (CPIs) and a large unmet need for new treatment options remains.

In recent years, small molecule antagonists that inhibit intracellular negative modulators of immune response have emerged as a novel class of therapeutics for cancer immunotherapy (CIT) (1). These molecules either act directly on targets that are part of negative feedback loops of immune signaling cascades, or they transmit immunosuppressive signals present within the tumor microenvironment (TME). By inhibiting these novel targets, the aim is to induce or enhance the anti-tumor immune response, deepen or sustain the response to checkpoint inhibition, and break primary or acquired resistance to CPI.

Various target classes and key therapeutic targets in adaptive and innate immunity came into focus: proteins that are part of negative feedback loops in T cell receptor (TCR) signaling, acting via their enzymatic function as a kinase, E3 ligase, or phosphatase; proteins involved in innate immunity signaling by negatively regulating the stimulator of interferon genes (STING) pathway; and receptors that transmit immunosuppressive signals caused by extracellular adenosine and prostaglandin E2 (PGE2).

We highlight the status of small molecule compounds in preclinical and clinical development that target these protein classes and address potential combination partners and tumor types in which these compounds are assessed. In addition, the identification of meaningful biomarkers is an increasingly important factor in the development of any new cancer medicine. Pharmacodynamic biomarkers to monitor target specific engagement or immuno-modulation in the clinic are being evaluated. A challenge for most targets is to identify biomarkers that can predict therapeutic responses, with the aim of enriching the target patient population for the respective indication.

## Small molecules targeting kinases and ubiquitin ligases involved in TCR signaling

### MAP4K1

The kinase MAP4K1 (mitogen-activated protein kinase kinase kinase kinase 1)/HPK1 (hematopoietic progenitor kinase 1) was one of the first drug targets identified for a CIT approach using small molecules (2). MAP4K1 is a serine/threonine kinase expressed predominantly in hematopoietic lineages with highest expression levels found in T cells, B cells, and dendritic cells (DCs) (3). The immunosuppressive potential of MAP4K1 is conferred primarily by its role as a negative regulator of the TCR signaling pathway via a negative feedback loop, as well as by its contribution to T cell suppression induced by extracellular adenosine levels, PGE2, and transforming growth factor beta (TGFβ) (2, 4, 5).

**Figure 1:**
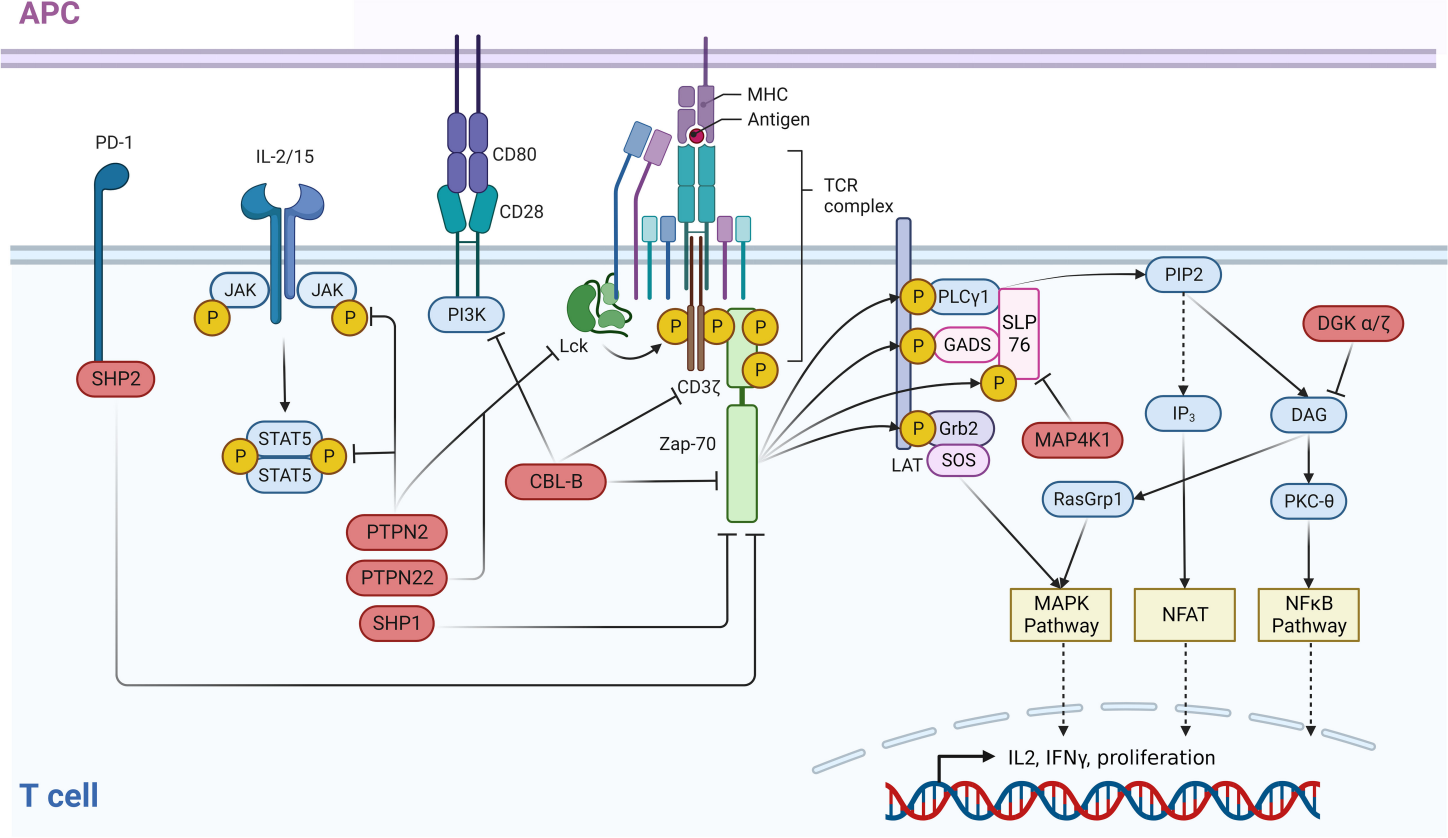
Negative regulators of the TCR and associated immune pathways. An intricate network of negative regulators ensures that signals contributing to the activation of T cells are correctly terminated after a certain period of active signaling. In the case of the TCR, following receptor binding to MHC-bound ligands, receptor distal signaling is down-modulated through a negative feedback loop by PTPN2 and PTPN22 which dephosphorylate LCK. ZAP70 activity is controlled by SHP-2 which dephosphorylates this kinase and CBL-B which ubiquitinates ZAP70. In parallel, CBL-B directly ubiquitinates and inactivates the TCR itself. In addition to this function, CBL-B negatively impacts the CD28 costimulatory pathway by ubiquitinating the PI3K subunit p85. Receptor distal negative regulators are MAP4K1, which impairs the TCR pathway via phosphorylation of SLP76, and DGKα/ζ. The latter eliminates the PKC and RasGRP1 activating ligand DAG via phosphorylation. T cell activating cytokine signaling via the JAK/STAT pathway is negatively regulated by the phosphatase PTPN2 which dephosphorylates both JAK family proteins and STAT5. This figure was created with BioRender.com.

In the TCR signaling pathway, MAP4K1 is activated by three different phosphorylation steps subsequent to its recruitment to the plasma membrane. Following TCR activation, zeta chain-associated protein kinase 70 (ZAP70) phosphorylates MAP4K1 on tyrosine 381, which enables interaction with the MAP4K1 substrate protein, Src homology 2 (SH2) domain containing leukocyte protein of 76 kDa (SLP76) (6). This initial phosphorylation step is followed by phosphorylation at serine 171 via protein kinase D1 (PKD1) and an auto-phosphorylation event at threonine 165 resulting in full MAP4K1 activation (7–9). Ultimately, MAP4K1 phosphorylates its substrate SLP76 at serine 376 which leads to binding of 14-3-3 proteins to SLP76, thereby disrupting effective downstream signaling (**Figure 1**) (10).

The MAP4K1-mediated negative regulation of T cell responses was studied in knockout mice, while the importance of its enzymatic function was elucidated using kinase-dead knock-in models (11–13). Following TCR signaling activation, *Map4k1* ^-/-^ mice respond with increased cytokine production and proliferation (11). Increased autoimmune responses were observed *in vivo* in an experimental autoimmune encephalomyelitis model (11). Enhanced *in vitro* and *in vivo* T cell responses were also described for *Map4k1* ^KD/KD^ kinase-dead transgenic mice, indicating that the enzymatic function of MAP4K1 is essential for its immunosuppressive function (12, 13). Bone marrow-derived antigen-presenting DCs from *Map4k1* ^KD/KD^ mice induced a stronger activation of wildtype T cells specific for the antigen, indicating that the enhanced tumor rejection in *Map4k1* ^KD/KD^ mice is a result of both stronger effector function and more efficient antigen cross-presentation (13).

Interestingly, *Slp76^SA/SA^* transgenic mice (mutant for the most studied MAP4K1 phosphorylation site, SLP76 serine 376) display a less pronounced phenotype with modulation of T helper cell (Th)1/Th2 cytokine production as the only overt difference to the wildtype. These findings indicate that phosphorylation of yet unidentified MAP4K1 substrates might contribute to the strong effect of MAP4K1 inactivation or deletion on T cell activation (14).

In syngeneic tumor models, *Map4k1* deficiency leads to rejection of intravenously injected Lewis lung carcinoma cells (LLC) (2). The key experiments validating MAP4K1 as a target for novel kinase inhibitors in CIT were generated by Hernandez et al. and Wang et al., who observed that tumor growth is inhibited in kinase-dead *Map4k1^KD/KD^* transgenic mice (12, 13). The *in vivo* tumor growth inhibition observed in cancer models of various cellular origin (GL261 glioma, MC38 colon adenocarcinoma, and 1956 sarcoma) supports the broad potential application of a MAP4K1 inhibitor (12, 13).

To mimic the effects of genetic inactivation of MAP4K1, various small molecule modalities were pursued, with ATP competitive kinase inhibitors being the most common (3). The most advanced MAP4K1 inhibitors are in early clinical development, with compounds from Nimbus Therapeutics, Treadwell Therapeutics, Pfizer, and BeiGene, being currently tested in phase 1 or combined phase 1/2 studies.

Nimbus Therapeutics’ NDI-101150 is an ATP competitive MAP4K1 inhibitor with a biochemical IC_50_ below 1 nM and an *in vivo* IC_50_ of 8.65 mg/kg, based on pSLP76 measurements from re-stimulated murine splenic T cells (15). NDI-101150 is highly selective against other members of the MAP4K family and spares kinases essential for T cell activation (15). *In vitro* experiments using exhausted T cells showed that pro-inflammatory cytokines are increased following treatment with NDI-101150, an effect that can be boosted by co-treatment with an anti-PD-1 antibody (15). *In vivo* testing of NDI-111050 in syngeneic efficacy models showed responses in colon adenocarcinoma (CT26), lymphoma (A20), breast cancer (EMT-6), and liver cancer models (Hepa1-6). Consistent with the *in vitro* findings, the combination of NDI-111050 with an anti-PD-1 antibody significantly improved survival relative to the anti-PD-1 single agent treatment in a CT26 efficacy model (15). The chemical structure of NDI-101150 is undisclosed, but it is very likely similar to example I-75 chosen from a recent Nimbus Therapeutics patent (**Table 1A**). Clinical development of NDI-101150 was initiated in 2021 with a phase 1/2 study testing safety, tolerability, pharmacokinetics, pharmacodynamics, and preliminary efficacy as a single agent or in combination with pembrolizumab in patients with advanced solid malignancies (NCT05128487). Limited information from this trial has been disclosed until now, but the company stated that responses were observed in CPI-refractory patients treated with NDI-101155.

**Table 1:** MAP4K1 inhibitors.

Compound	Target	Indication/Combination/ Preclinical model	Phase	Company	Reference
NDI-101150 (NMBS-2)	MAP4K1	Solid tumors (expansion cohorts in gastric and GEJ* cancer patients) (+ pembrolizumab)	1	Nimbus Therapeutics	NCT05128487
Patent Example I-75 (A)	MAP4K1	–	Preclinical	Nimbus Therapeutics	WO2022/187856
CFI-402411	MAP4K1	Solid tumors (+ pembrolizumab)	1/2	Treadwell Therapeutics	NCT04521413
Patent Example 5 (B)	MAP4K1	–	–	Treadwell Therapeutics	WO2021/226707
PF-07265028	MAP4K1	Advanced solid tumors (+ sasanlimab)	1	Pfizer	NCT05233436
Compound 21 (C)	MAP4K1	–	Preclinical	Pfizer	(16)
AZ1/Patent example 213 (D)	MAP4K1	CT26, MCA205	Preclinical	AstraZeneca	WO2023/001794
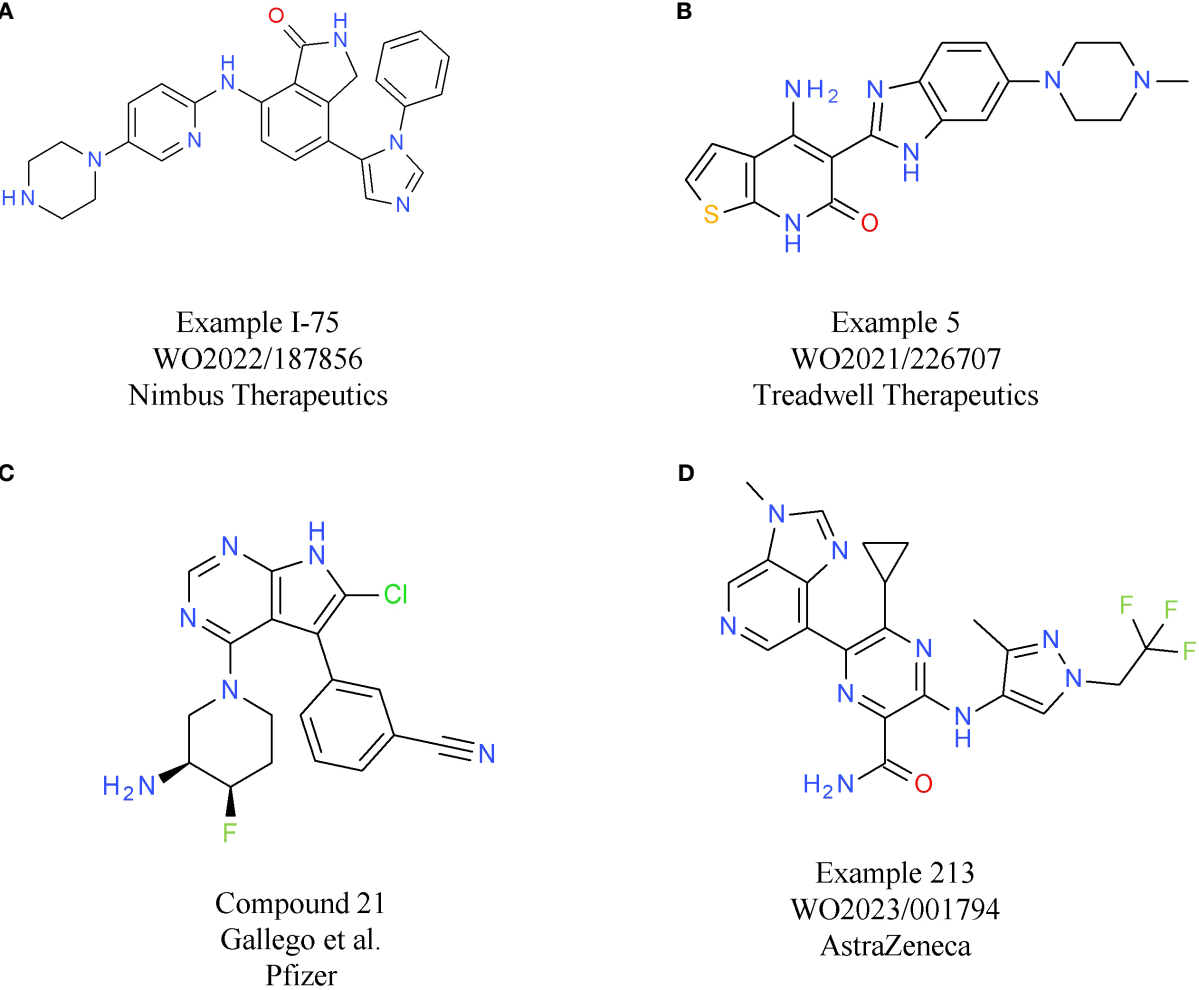					

*GEJ, gastroesophageal junction.

CFI-402411 from Treadwell Therapeutics Inc. is the first MAP4K1 small molecule inhibitor for which clinical data have been recently shared publicly (17, 18). CFI-402411 is currently in a phase 1/2 dose-escalation study (study TWT-101) in combination with pembrolizumab (NCT05128487) (18)). The aim of the phase 1/2 study is to determine the maximum tolerated dose (MTD) and the recommended phase 2 dose (RP2D) for CFI-402411 as a single agent and in combination with pembrolizumab, respectively. In 2022, data for 31 patients with a wide range of CPI treatment-sensitive solid cancers were published. Promising efficacy was observed for two patients with head and neck squamous cell carcinoma (HNSCC), who showed partial responses. One response was observed in the single agent CFI-402411 arm (35% tumor size reduction) and one response, with a more pronounced tumor shrinkage, was observed in the CFI-402411 and pembrolizumab combination arm (81% lesion size reduction). Both patients with partial responses were previously treated with pembrolizumab. For 9/32 patients the best response was stable disease. CFI-402411 has a clinically manageable safety profile, with the most common toxicities being diarrhea, fatigue, and nausea. Patient exposure data and pharmacokinetic/pharmacodynamic modeling indicate that in the highest dose cohorts, CFI-402411 inhibits MAP4K1 enzymatic activity for 24 hours per day. The chemical structure of CFI-402411 is currently unknown. In **Table 1B** an example with attractive *in vitro* properties from a recent Treadwell patent is depicted, offering some insight into the focus of medicinal chemistry efforts at Treadwell.

Clinical development of a MAP4K1 inhibitor has also been initiated recently by Pfizer. PF-07265028 will be assessed either as monotherapy or in combination with the anti-PD-1 antibody sasanlimab in 240 patients with advanced or metastatic solid tumors in Japan and the United States (NCT05233436). The key indication pursued in this study is head and neck cancer. The aim of this study is to evaluate safety, tolerability, pharmacokinetics, and pharmacodynamics as well as to determine the MTD and RP2D. The chemical structure of PF-07265028 has not been disclosed to date, but a recent publication provides some insight into compound design for a MAP4K1 inhibitor (16). Compound optimization from a spirocyclic molecule identified in a virtual screen focused mainly on gaining selectivity over kinases involved in TCR signaling. In fact, the starting point was such an efficient PKCθ inhibitor that TCR responses to ligand stimulation were fully blocked. Following optimization processes to eliminate PKCθ activity, compounds were designed to be selective towards other members of the MAP4K family (MAP4K2/3/5) and MST1/2. After improving selectivity over off-target kinases, medicinal chemistry efforts focused on increasing potency and improving drug-like properties, ultimately leading to compound 21 (**Table 1C**). It is currently unknown if the scaffold described in this publication was further optimized to the clinical candidate PF-07265028. The primary completion date of the phase 1 study is January 2026.

Clinical trials are underway for two other molecules: BeiGene’s BGB-15025 and Zhuhai Yufan Biotechnologies’ PRJ1-3024 (structures undisclosed). Similar to the molecules described above, BeiGene’s MAP4K1 inhibitor increases T cell responses and causes tumor growth inhibition in syngeneic mouse models (GL261, CT26, and EMT6 breast cancer). Like many other companies, BeiGene is developing BGB-15025 in combination with immune checkpoint blockade, namely the proprietary anti-PD-1 antibody tislelizumab (19).

Two MAP4K1 inhibitors close to reaching clinical development are AZ1 from AstraZeneca and BLU-852 from Blueprint Medicines. AZ1 (example 213, **Table 1D**) increases the response of stimulated T cells. At the cytokine level, gene expression analysis shows that the main signaling pathways with enhanced activity following treatment with AZ1 are the nuclear factor kappa B (NFκB) and MAPK pathways (20). *In vitro*, AZ1 treatment rescued T cell exhaustion and restored T cell effector functions. *In vivo*, AZ1 was efficacious in MCA205 fibrosarcoma and CT26 adenocarcinoma syngeneic tumor models. Robust efficacy was achieved with an average of 90% inhibition of the pharmacodynamic marker pSLP76. Details on the clinical development of AZ1 are currently not available, but it can be expected that the molecule will be tested in patients within a reasonable timeframe.

For BLU-852, which was discovered in collaboration between Blueprint Medicines and Roche, preclinical data were shared at AACR 2021. BLU-852 is a MAP4K1 inhibitor with subnanomolar activity in a biochemical assay and has more than 500-fold selectivity over the key off-target kinases lymphocyte-specific protein tyrosine kinase (LCK) and MAP4K4 (21). The overall selectivity of BLU-852 is high, with more than 100-fold selectivity over 97% of the kinome in a binding assay. Cellular activity of BLU-852 was demonstrated through increased interleukin 2 (IL2) production in stimulated T cells, and inhibition of pSLP76 phosphorylation confirmed target suppression in T cells. *In vivo* data for BLU-852 are not available yet, but for two additional molecules disclosed on the poster single agent activity was evident in MCA205, EMT6, and MC38 syngeneic efficacy models. Efficacy was dependent on CD8^+^ T cells, linked to intratumoral immune-modulation, and could be enhanced by combination with an anti-PD-L1 antibody (atezolizumab mouse surrogate).

In the preclinical space, a very high activity with regard to MAP4K1 inhibitors was reported in recent years from Merck & Co., Genentech, Bristol-Myers Squibb, Janssen, as well as several other biotech companies (22–24). Clinical data expected within the next 2-3 years will give further insight into the potential of the compounds described above to materialize into novel CIT drugs.

### DGKα/ζ

DGKα and DGKζ belong to the family of diacylglycerol (DAG) kinases that comprises 10 lipid kinases, all phosphorylating the second messenger DAG (25). DGKα is exclusively expressed in T cells, whereas the expression of DGKζ is restricted to hematopoietic lineages and the central nervous system. Both kinases are negative regulators of TCR signaling and are therefore targets of current drug discovery efforts (26).

In the TCR pathway, phospholipase C gamma (PLCγ) is activated after receptor ligation, cleaving phosphatidylinositol (4,5)-trisphosphate (PIP2) into inositol-1,4,5-trisphosphate (IP3) and DAG. DAG serves as a second messenger and its binding leads to activation of PKCθ and rat sarcoma virus guanyl releasing protein 1 (RASGRP1). Both proteins interact with DAG via their C1 domain and their activation induces downstream signaling events (27). Activated PKCθ phosphorylates CARD11 to induce CARD11-BCL10-MALT1 (CBM) complex formation, which triggers downstream signaling events that ultimately lead to nuclear translocation of the transcription factor NFκB (28). Activated RASGRP1 induces the MAPK pathway and triggers the transcription of AP1 target genes. Therefore, phosphorylation of DAG by DGKα/ζ reduces the levels of this second messenger resulting in attenuation of TCR signaling (**Figure 1**). Consequently, increased TCR responses have been reported for *Dgkα^-/-^* and *Dgkζ^-/-^* mice (29). In both cases, suboptimal stimulation of knockout T cells causes a stronger cytokine production and leads to more pronounced T cell proliferation compared to wildtype control cells. Thereby, DGKζ deletion induces stronger T cell activation across all readouts studied (29). Increased T cell responses were not limited to murine knockout T cells, but were also observed with other approaches, such as deleting DGKα, DGKζ, or both in human T cells or human chimeric antigen receptor (CAR)-T cells (29, 30). The adoptive transfer of DGKα/DGKζ-deleted CAR-T cells caused a compelling tumor rejection in a subcutaneous xenograft model. Consistent with the *in vivo* tumor growth inhibition in the adoptive transfer models, tumor growth in *Dgkα* or *Dgkζ* knockout mice was attenuated (29). Similar to the *in vitro* situation, deletion of Dgkζ caused more pronounced tumor growth inhibition in the syngeneic *in vivo* efficacy models MC38, B16 melanoma, and C1498 leukemia. In line with this, superior tumor control in Dgkζ^-/-^ mice correlated with a larger fraction of activated T cells (29).

Based on the convincing effects observed by genetic inactivation of DGKα and/or DGKζ, several companies started working on DGKα/ζ dual or paralog-selective small molecule inhibitors, although data on kinase inactive knock-in mice were not available at that time. So far, the most comprehensive data on DGK inhibitors have been released from Bristol-Myers Squibb and Bayer.

Bristol-Myers Squibb has disclosed molecules at symposia and in patents that are related to the first identified weak DGKα inhibitor ritanserin (**Table 2A**). Ritanserin was originally discovered as a 5-HT2A receptor antagonist (31). Despite some similarity to ritanserin, the naphthyridinone series from Bristol-Myers Squibb was identified through various parallel phenotypic T cell screens (32). For one hit, a quinolone (compound 1/BMS-684, **Table 2B**), the respective targets were identified as DGKα and DGKζ. Optimization of the series resulted in molecules with potent activity in enzymatic assays using lipid substrates (33). Currently, the most advanced compounds have submicromolar activity on DGKα and DGKζ, such as compound 103 from a recent patent (**Table 2C**) (33). Most of the disclosed compounds target DGKα and DGKζ and largely spare the other kinases of the DGK family (32). The *in vitro* and *in vivo* properties of one compound were presented at a recent symposium (34). Treatment of T cells with the DGKα/ζ inhibitor lowered the TCR activation threshold. Especially under conditions of suboptimal T cell priming, DGKα/ζ inhibitor treatment increased T cell cytokine production (34). To understand if DGKα/ζ inhibition can synergize with immune checkpoint blockade *in vitro*, mixed lymphocyte reaction assays were conducted in the presence of the DGKα/ζ inhibitor, nivolumab, or both. Single agent anti-PD-1 antibody and DGKα/ζ inhibitor treatment caused elevated interferon gamma (IFNγ) levels, while their combination resulted in the most efficient induction of cytokine production. *In vivo*, the same additive effect between anti-PD-1 antibody and DGKα/ζ inhibitor treatment was evident in the MC38 model. In addition, in the T cell-excluded melanoma model B16, compelling effects were observed, with DGKα/ζ inhibitor treatment prolonging overall survival. Overall, the data showed that the effects obtained with DGKα/ζ inhibitors are very similar to what has been reported for the *Dgkα* and *Dgkζ* knockout mice. Clinical development timelines and structures of clinical candidates have not been disclosed to date by Bristol-Myers Squibb.

**Table 2:** DGKα/ζ inhibitors.

Compound	Target	Indication/ Combination/ Preclinical model	Phase	Company	Reference
Ritanserin (A)	DGKα (serotonin receptor antagonist)	–	–	Janssen Pharmaceutical	(31)
Compound 1 (BMS-684) (B)	DGKα/ζ	–	Preclinical (HTS hit)	Bristol-Myers Squibb	(32)
Compound 103 (C)	DGKα/ζ	–	Preclinical	Bristol-Myers Squibb	WO2020006018 (33)
BAY-2965501(D)	DGKζ	Advanced solid tumors	1	Bayer Pharmaceuticals	NCT05614102
BAY-2862789	DGKα	Advanced solid tumors (NSCLC)	1	Bayer Pharmaceuticals	NCT05858164
Patent example 539 (E)	DGKα	–	–	Bayer Pharmaceuticals	WO2021/105117
ASP-1570	DGKζ	Advanced solid tumors (+ pembrolizumab)	1	Astellas Pharmaceuticals	NCT05083481
Patent example 1 (F)	DGKζ	B16F1	Preclinical	Astellas Pharmaceuticals	WO2022/114164
GS-9911	DGKα	–	Preclinical	Gilead Sciences/Carna	Gilead Oncology Deep Dive Presentation 2022
BGB-30813	DGKζ	Advanced solid tumors (+ tislelizumab)	1	BeiGene	NCT04649385
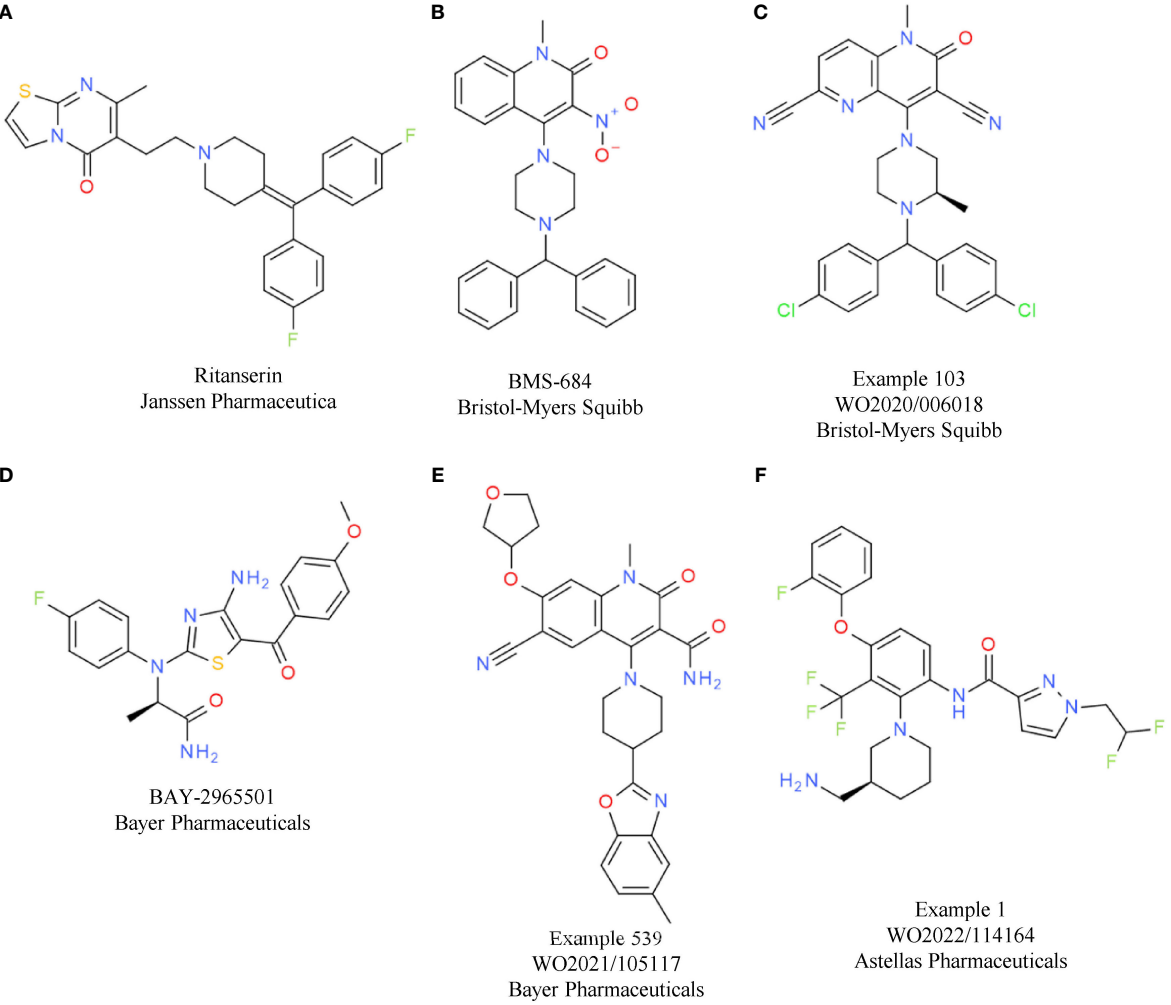					

In contrast, the structure of the DGKζ inhibitor BAY-2965501 from Bayer was recently presented (35). BAY-2965501 has submicromolar activity on DGKζ and an ATP-cooperative binding mode (**Table 2D**). It is unclear if BAY-2965501 has activity on DGKα, but since Bayer’s pipeline also lists a DGKα inhibitor, it is likely that a paralog-selective approach was prioritized over dual DGKα/ζ targeting. *In vitro*, BAY-2965501 treatment resulted in enhanced cytokine production from T cells and increased cytotoxic activity. Moreover, BAY-2965501 treatment could counteract the immunosuppressive potential of adenosine and PGE2 signaling on T cells (35). In an F9 testicular teratoma model, BAY-2965501 showed a dose-dependent tumor growth inhibition, with 9/12 mice in the highest dose cohort being tumor-free after treatment. The combination of BAY-2965501 with immune checkpoint blockade resulted in additive or synergistic effects. Consistent with the strongest tumor growth inhibition being observed in the combination groups, the increase of intratumoral T cell responses was most pronounced when DGKζ inhibition was combined with checkpoint blockade. BAY-2965501 has an acceptable safety profile with no findings in cardiovascular studies and central nervous system assessments, which were of special interest due to DGKζ’s expression in brain tissues. BAY-2965501 is being investigated in a first-in-human clinical study to assess its pharmacokinetics, safety, and preliminary efficacy, and determine the MTD and RP2D (NCT05614102). Patients with advanced solid tumors are being enrolled in this study, with the expansion part focusing on non-small cell lung cancer (NSCLC) and gastric/gastroesophageal junction adenocarcinoma. CPI combination plans have not been reported.

A second DGK inhibitor from Bayer that has progressed to the clinic is BAY-2862789, which, unlike BAY-2965501, is a DGKα paralog-selective molecule. To date, the chemical structure of BAY-2862789 is undisclosed, but recent patents suggest that Bayer focused on a ritanserin-related scaffold (WO 2021105115/6/7). Example molecules from the patent possess submicromolar activity in the mixed micelle ADP-glo assay; a representative compound (example 539) is shown in **Table 2E**. In contrast to the DGKζ series, further preclinical information on the DGKα targeting molecules has not been disclosed. BAY-2862789 is being investigated in a phase 1 study to assess its safety, pharmacokinetics, and preliminary efficacy and determine the MTD and RP2D. Similar to BAY-2965501, the study includes only a single agent arm. The indication for clinical development of BAY-2965501 is NSCLC (NCT05858164).

Based on current patent information, ASP-1570 from Astellas Pharma is very likely based on a scaffold similar to that of BAY-2965501. A representative example from a recent patent is depicted in **Table 2F**. ASP-1570 is a DGKζ selective inhibitor that can restore T cell function under immunosuppressive conditions (PGE2, adenosine, TGFβ). In syngeneic efficacy models, ASP-1570 caused tumor growth inhibition in MC38 and B16F1 models (36). Overall, ASP1570 treatment led to effects similar to genetic inactivation of DGKζ. The ongoing phase 1/2 dose-escalation studies are assessing safety, pharmacokinetics, and efficacy in patients with NSCLC and melanoma. In the phase 1/2 study, ASP-1570 will be studied as a monotherapy and in combination with pembrolizumab (NCT05083481).

Additional molecules in preclinical or early clinical development include GS-9911 and BGB-30813, identified by Gilead Sciences/Carna Biosciences and BeiGene, respectively. GS-9911 is a DGKα-selective molecule listed in the Gilead pipeline that was in-licensed from Carna Biosciences in 2019. BGB-30813 is a DGKζ inhibitor in early clinical development in combination with BeiGene’s anti-PD-1 antibody tislelizumab (NCT5904496).

In summary, dual DGKα/ζ inhibitors as well as DGKα or DGKζ – selective inhibitors are close to or currently in clinical development. Whether the dual or paralogue-selective approach will be the more successful path forwards currently is challenging to predict. Based on the preclinical data, the dual DGKα/ζ inhibition results in the stronger effects on immuno-modulation though. The first clinical data for some assessment of DGKζ inhibitors for CIT will most likely be released by Bayer (phase 1 primary completion for DGKζ inhibitor BAY-2965501 is in 2026).

### CBL-B

The ubiquitin E3 ligase CBL-B is a negative regulator of TCR signaling via its enzymatic function and an attractive target for small molecule inhibitors based on compelling *in vitro* and *in vivo* data. CBL-B belongs to the family of Casitas B-lineage lymphoma (CBL) proteins, which contain a catalytic RING finger domain and a regulatory tyrosine kinase binding (TKB) domain (37). CBL-B is expressed mainly in hematopoietic lineages, while the closely related c-CBL is expressed ubiquitously. Nonetheless, genetic deletion of either CBL-B or c-CBL leads to increased T cell activation after antigen stimulation (38). Two substrates ubiquitinated by CBL-B, in complex with additional proteins, are the p85 subunit of phosphoinositide 3-kinase (PI3K) and the TCRζ chain. For both substrates, ubiquitination impairs TCR signaling in a proteolysis-independent manner (39–41) (**Figure 1**). The lower T cell activation threshold reported for *Cbl-b^-/-^* mice results in spontaneous tumor rejection in adoptive T cell transfer experiments using *Cbl-b* deficient polyclonal CD8^+^ T cells (42). The relevance of the enzymatic function of *CBL-B* was demonstrated by comparing phenotypes of *Cbl-b* deficient and E3 ligase-inactive *Cbl-b^CA/CA^* transgenic mice (43). Cytokine production and proliferation were comparable between *Cbl-b^-/-^* and *Cbl-b^CA/CA^* knock-in T cells. In both cases, the T cell responses were significantly higher than in the wildtype T cell controls. Deletion or mutation of *Cbl-b* resulted in spontaneous autoimmunity. Strikingly, tumor growth inhibition in a human papilloma virus-expressing TC1 subcutaneous efficacy model was also similar between *Cbl-b^CA/CA^* mice and *Cbl-b^-/-^* mice, indicating that the increase in T cell activity is mostly derived from its catalytic function (43).

Based on the characteristics of CBL-B as a negative modulator of T cell activity, drug discovery programs on CBL-B inhibitors have been initiated by several companies. To date, the most comprehensive data on their discovery efforts were released by Nurix Therapeutics. Nurix identified binders to CBL-B in multiple, parallel hit finding approaches including a high-throughput screening (HTS), a DNA-encoded library screen, and a fragment-based screen (44). Structural analyses of the compounds that were selected showed that they act as “intramolecular glues”, keeping CBL-B in its closed, inactive conformation. Further x-ray crystallography studies of compound 23/C7683 elucidated that the molecule binds to the tyrosine kinase binding (TKB) and linker helix region (LHR) domains, while sparing the catalytic RING domain (**Table 3A**) (45). In T cell assays, the clinical candidate NX-1607 from Nurix increased IL2 and IFNγ production (46). The structure of NX-1607 has not been formally disclosed, but it is very likely similar or identical to compound 23/C7683. In syngeneic efficacy models, promising tumor growth inhibition was evident with NX-1607 in three different tumor models. Depleting either CD8^+^ T cells or NK cells impaired the efficacy of NX-1607, confirming the immuno-modulatory mechanism of action of the drug candidate. The combination of NX-1607 and an anti-PD-1 antibody increased tumor growth inhibition and median overall survival in all mouse efficacy models tested (46). Despite the compelling activity of the combination with the anti-PD-1 antibody, Nurix advanced NX-1607 into clinical phase 1 trials without CPIs as combinations partners (47). It can be speculated that after assessing pharmacokinetics/pharmacodynamics, tolerability, safety, and recommended phase 1b dose (RP1bD), Nurix might evaluate the combination with a CPI in later stages of development. NX-1607 is being assessed in tumor types known to be responsive to checkpoint blockade (NCT05107674). Interestingly, NX-1607 is also tested in diffuse large B cell lymphoma (DLBCL, post-Richter transformation), which is supported by preclinical data showing increased rituximab antibody-dependent cellular cytotoxicity-driven efficacy by CBL-B inhibition (48). Dose escalation data suggest a dose-proportional increase in exposure of NX-1607.

**Table 3:** E3 ligase CBL-B inhibitors.

Compound	Target	Indication/Combination/ Preclinical model	Phase	Company	Reference
NX-1607	CBL-B	Advanced malignancies (solid tumors and DLBCL) (+ paclitaxel for selected indications)	1	Nurix Therapeutics	NCT05107674
Example 23/C7683 (A)	CBL-B	–	–	Nurix Therapeutics	WO2020/264398 (45)
HST-1011	CBL-B	Advanced malignancies (+ cemiplimab)	1/2	HotSpot Therapeutics	NCT05662397
Example 10 (B)	CBL-B	–	–	HotSpot Therapeutics	WO2022/221704
Example 62 (C)	CBL-B	–	Preclinical	Nimbus Therapeutics	WO2022/217276
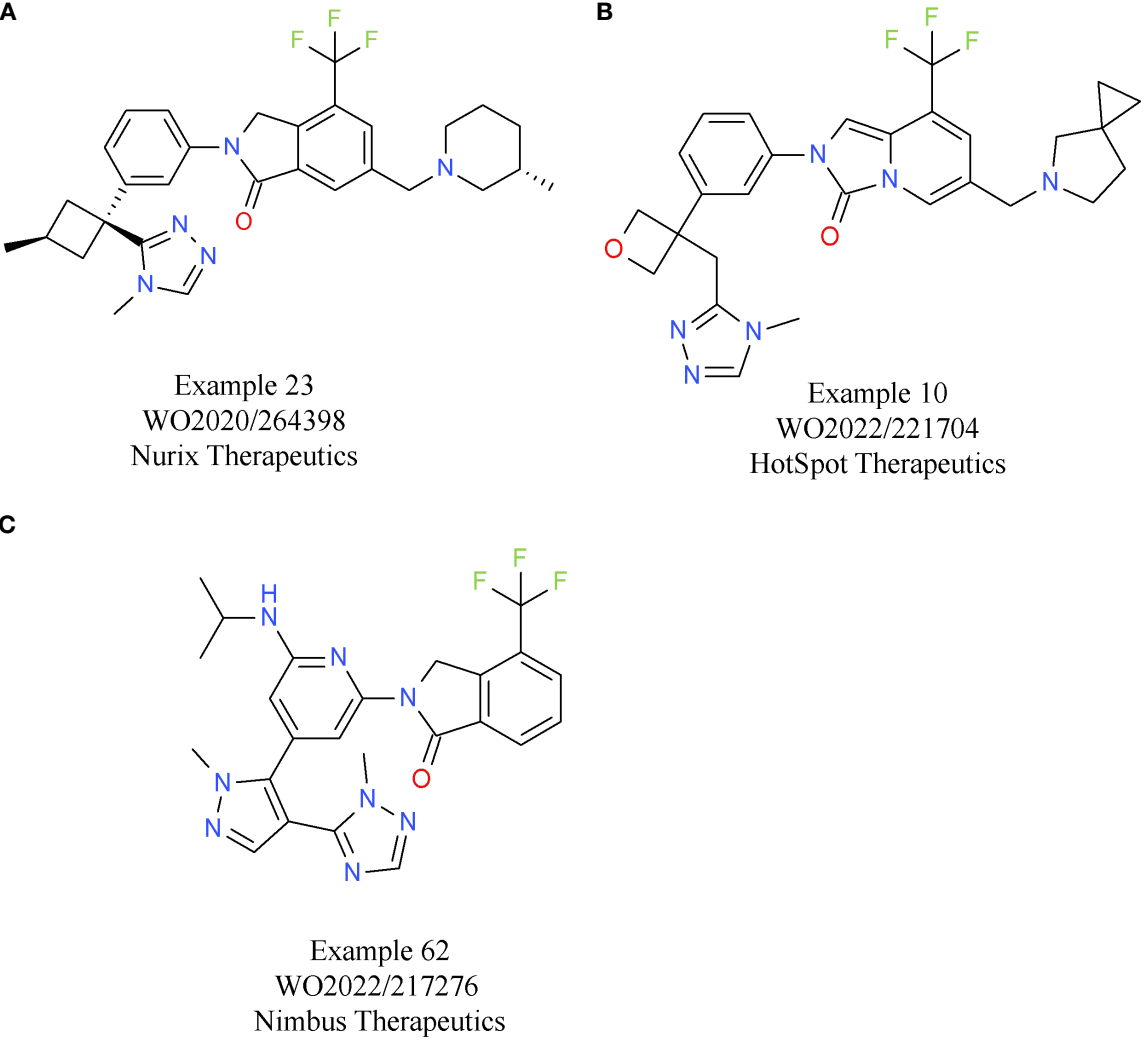					

The lead allosteric CBL-B inhibitor of HotSpot Therapeutics, HST-1011, entered the clinical development phase in late 2022. HotSpot did not publicly disclose the structure of HST-1011, but *in vitro* and *in vivo* preclinical data and clinical data were shared at different symposia. An example from a recent HotSpot patent is depicted in **Table 3B**, showing that this compound series is related to the scaffold that Nurix is focusing on. *In vitro*, HotSpot’s HOT-A/HST-1011 increases cytokine production from treated naïve and exhausted T cells, respectively (49). Elevated T cell responses result in tumor growth inhibition in a syngeneic CT26 model (49). In the ongoing phase 1/2 study, HST-1011 is given as monotherapy and in combination with an anti-PD-1 antibody (NCT05662397). Patients with solid tumors who either relapsed on, or are refractory to, anti-PD-(L)1 therapies are treated with increasing doses of HST-1011. Following dose escalation as monotherapy, combination arms with the anti-PD-1 antibody cemiplimab will be initiated (50).

Preclinical data of the CBL-B inhibitor of Nimbus Therapeutics have been presented at various scientific conferences. NTX-001 blocks CBL-B, resulting in enhanced phosphorylation of ZAP70 and consequently, generally enhanced T cell responses. *In vivo* data of NTX-001 are limited to studies in the CT26 efficacy model where the compound has single agent activity (51). The currently available patent information indicates that Nimbus CBL-B inhibitors rely on a chemical scaffold very similar to that from HotSpot and Nurix Therapeutics. An example from a recent Nimbus patent is shown in **Table 3C**.

In summary, multiple, chemically similar CBL-B inhibitors have been generated with the most advanced compounds being in early clinical trials. The selectivity of these molecules over c-CBL is currently unknown. Based on the available protein sequence and structural information, all compounds should have similar potency for CBL-b and c-CBL (45). Since the double knockout of *Cbl-b* and *c-Cbl* in mice results in embryonic lethality (52), it will be interesting to see if the generated compounds have a sufficiently large therapeutic window.

## Small molecules targeting phosphatases involved in the innate and adaptive immune responses

### PTPN2

The non-receptor protein tyrosine phosphatase TC-PTP, encoded by the *PTPN2* gene, is expressed in humans as two isoforms of 45 and 48 kDa, respectively, generated by alternative splicing. TC45, the main isoform, translocates between the nucleus and cytoplasm in response to cellular stimuli, while TC48 is located in the endoplasmic reticulum (ER) (53, 54). At a functional level, TC-PTP inhibits signaling downstream of pro-inflammatory cytokine receptors by dephosphorylating and inhibiting janus kinases (JAKs) and signal transducer and activator of transcription proteins (STATs), and regulates TCR signaling by dephosphorylating Src family kinase (SFK) activation motifs. Growth factor receptors such as epidermal, platelet-derived, and vascular endothelial growth factor receptors (EGFR, PDGFR and VEGFR) are regulated by PTPN2 (55). PTPN2 activity is regulated allosterically through its disordered C-terminal tail, which keeps the protein in an inhibited conformation. Displacement of this auto-inhibitory region results in TC45 activation (56, 57).

Experiments in mice showed that *Ptpn2* deletion enhances immune-mediated tumor control by sensitizing tumors to cytotoxic T cell killing and enhancing anti-tumor responses of T cells. *In vivo*, PTPN2 deletion results in increased anti-tumor immunity, with *Ptpn2* deficient CD8^+^ T cells displaying better cytotoxic activity and reduced exhaustion compared to wildtype cells (58). Transplanted *Ptpn2^-/-^* T cells better protect older *Tp53* ^+/−^ mice from developing tumors and hinder the growth of transplanted mammary cancer, resulting in an increased presence of activated CD4^+^ and CD8^+^ effector and memory T cells in the tumor (59). Moreover, in an adoptive transfer setting, *Ptpn2*-deficient T cells inhibit tumor growth, and in CAR-T cells a lack or inhibition of PTPN2 fosters the production, antigen-specific activation, and cytotoxicity of CD8^+^ HER2 CAR-T cells *ex vivo*, while reducing HER2^+^ E0771 mammary tumor growth. In mice treated with *Ptpn2^-/-^* CAR-T cells, tumor control is achieved without autoimmunity and severe morbidity for up to 70 days post-transfer (59). In line with the findings described above, *Ptpn2* knockout in hematopoietic cells results in complete clearance of MC38 and B16 tumors and in a more effective response to GVAX-anti-PD-1 immunotherapy (58). In an *in vivo* study using CRISPR-Cas9 in transplanted B16 melanoma tumors in mice, *Ptpn2* deletion in cancer cells has been shown to improve tumor response to immunotherapy, including an anti-PD-1 antibody and a tumor cell vaccine (GVAX) (60). *Ptpn2* knockdown likely increases melanoma cell sensitivity to IFNγ by enhancing STAT1, STAT3, and STAT5 phosphorylation and MHC-I, MHC-II, and PD-L1 expression (61, 62). Consistent with the above results, the combined deletion of PTPN2 in T cells and cancer cells acts in a synergistic way by fostering T cells recruitment and activation resulting in a further repressed tumor growth (63).

Despite significant sequence similarity among the PTP superfamily proteins, selective small molecule inhibitors targeting the phosphatase activity of the related proteins PTP1B and SHP2/PTPN11 have been successfully generated by utilizing subtle sequence differences in the outer region of the catalytic domain (64, 65). Two orally available active site dual PTPN2/PTP1B inhibitors, ABBV-CLS-579 (**Table 4A**) and ABBV-CLS-484 (**Table 4B**; patent publication WO2019/246513) are in phase 1 clinical trials for advanced or metastatic tumors (NCT04777994, NCT04417465). However, due to the high similarity between PTPN2 and PTP1B, particularly in the catalytic site, identifying PTPN2 selective molecules with suitable pharmacological profiles has been challenging (57, 66). Nevertheless, since PTP1B deficiency or inhibition has also been reported to hinder the growth of various tumors through enhancement of T cell-mediated anti-tumor responses (67), strict selectivity over PTP1B may not be required, and limited selectivity could even enhance therapeutic efficacy (68). Dual PTPN2/PTP1B proteolysis targeting chimera (PROTAC) inhibitors, which lead to selective degradation of PTPN2 and PTP1B, have been also developed by Abbvie/Calico and are tested preclinically (WO2021127586). Given that PTPN2 is regulated allosterically, selective targeting outside the catalytic domain may be possible (56, 57), although this has not been proven yet.

**Table 4:** Phosphatase antagonists PTPN2, PTPN22, SHP1/2.

Compound	Target	Indication/ Combination/ Preclinical model	Phase	Company/ Developer	Reference
ABBV-CLS-579 (A)	PTPN1/2	HNSCC, NSCLC, ccRCC*	1	AbbVie/Calico	WO2019/246513 NCT04417465
ABBV-CLS-484 (B)	PTPN1/2	HNSCC, NSCLC, ccRCC*	1	AbbVie/Calico	WO2019/246513 NCT04777994
L-1 (C)	PTPN22	MC38, CT26	Preclinical	Zhang Laboratory, Purdue University	WO2021/007491
TPI-1 (D)	SHP1	B16, K1735*, MC-26*	Preclinical	Taolin Yi, Taussig Cancer Center	PMID: 20421638
SHP099 (E)	SHP-2	CT26, MC38, 4T1	Preclinical	Novartis	PMID: 27362227 PMID: 27347692 WO2015/107493
TNO155 (F)	SHP-2	NSCLC, SCC, Head/Neck SCC, Melanoma	1 and 1/2	Novartis	NCT03114319 NCT04000529 NCT04330664 NCT04294160 NCT04292119 NCT04185883 NCT04699188 WO2015/107493
RMC-4630 (G)	SHP-2	PAAD, CRC*, NSCLC, KRAS mutated tumors	1 and 1/2	Revolution Medicines	NCT03634982 NCT04916236 NCT03989115 NCT04185883 NCT04418661 WO2020/247643
BBP-398 (H)	SHP-2	NSCLC	1	BridgeBio/Bristol-Myers Squibb	NCT04528836 NCT05375084 NCT05480865 WO2017/210134
JAB-3068/JAB-3312 (I, J)	SHP-2	NSCLC, HNSCC, ESCA*, CRC, PDAC*, BC*	1/2	Jacobio	NCT03518554 NCT03565003 NCT04721223 NCT04045496 NCT04121286 NCT04720976 WO2017/211303 WO2018/172984
RLY-1971 (K)	SHP-2	Advanced solid tumor	1	Relay Therapeutics/ Genentech	NCT04252339 WO2021/061706
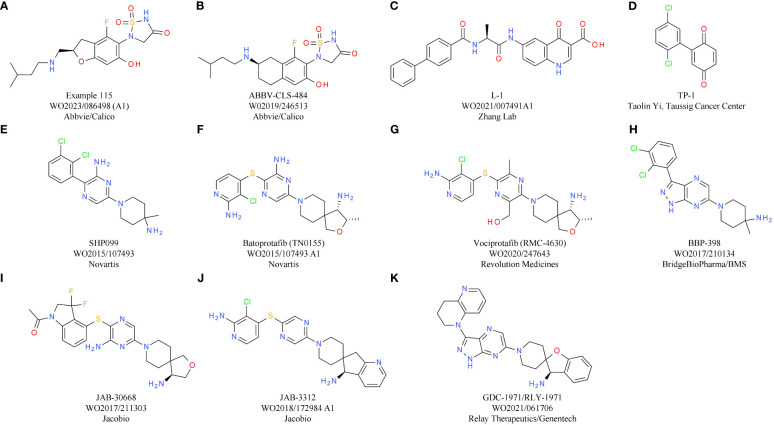					

*ccRCC, clear cell renal cell carcinoma.

*K1735, mouse melanoma cell line.

*MC-26, mouse colon cancer cell line.

*4T1, murine mammary carcinoma cell line.

*CRC, colorectal cancer.

*ESCA, esophageal cancer.

*PDAC, pancreatic ductal adenocarcinoma.

*BC, breast cancer.

### PTPN22

PTPN22 is a non-receptor protein tyrosine phosphatase preferentially expressed by hematopoietic cells and characterized by three distinct domains: an N-terminal PTP domain, an inter-domain, and a C-terminal domain with four proline rich sequence motifs that contribute to the interaction with the SH3 domain of the C-terminus of Src family kinase (CSK) (69). PTPN22 suppresses TCR signaling by removing phosphate groups from early signaling molecules such as SFKs, ZAP70, TCRζ, Vav, and valosin-containing proteins (70). Additional regulatory functions identified for PTPN22 include inhibition of regulatory T lymphocytes (Treg) cell immunosuppressive-related activities (71), IL6R signaling in B cells (72), NOD2 signaling in myeloid cells (73), DC dectin 1 signaling (74) and IFNα receptor signaling (75). Furthermore, PTPN22 enhances type 1 interferon responses following pattern recognition receptor activation in myeloid cells (76), activates inflammasomes by dephosphorylating NLRP3 in myeloid cells (77), and influences neutrophil effector functions (78) and mast cell IgE receptor signaling (79).

Based on its role in inhibiting early TCR signaling and consequently T cell activation, PTPN22 is considered a potential target for boosting T cell-mediated cancer immunity. The importance of PTPN22 in the regulation of immune-mediated tumor control is supported by mouse and human data. In humans, the *PTPN22* ^R620W^ variant is linked to autoimmunity and significantly associated with protection against various cancers and an enhanced response to CPI therapy (75, 80). Mice with an R619W knock-in transgene (which corresponds to the human autoimmune-associated R620W alteration) exhibit better control of xenografted Hepa1-6.x1 tumor growth than wildtype mice, but less effectively than *Ptpn22*-deficient mice (75, 81). *Ptpn22* ^R619W^ mutant mice show greater control of immunogenic B16-OVA and MC38 tumors, with increased tumor immune cell infiltration. However, in another study no significant differences were observed between *PTPN22* ^R619W^ mutant and wildtype mice in controlling poorly immunogenic LLC and B16-F10 tumor growth (81). Global *PTPN22* deletion enhances the growth inhibitory effects of anti-PD-L1 treatment on MC38 and CT26 tumors and results in an increased CD8^+^ T cell infiltration and CD8^+^ T cell to Treg cell ratio. Similar to CD8^+^ T cells, tumor-infiltrating macrophages and NK cells were also increased within tumors of Ptpn22^-/-^ mice (80). In line with the phenotype of *Ptpn22* ^-/-^ mice, adoptive transfer of *Ptpn22*-deficient OT-I T cells was shown to control the growth of OVA peptide-expressing lymphoma and ovarian carcinoma tumors more effectively and also enhance the efficacy of a TGFβ blocking antibody (82, 83). Contrary to this finding, a newer study reported that PTPN22 deficiency did not influence the effectiveness of adoptively transferred OT-I T cells against OVA peptide-expressing AT3 mammary or MC38 tumors, nor did it improve the performance of human or mouse CAR-T cells against solid tumors *in vivo*. This indicates that PTPN22 may not be an ideal target for adoptive T cell therapy (84).

PTPN22 is thought to regulate the observed anti-tumor immunity through its catalytic function based on the similar inhibition of Hepa1-6.x1 tumor growth observed with *Ptpn22* ^C227S^ inactive knock-in mice and PTPN22-deficient mice (75). Therefore, the development of small molecule therapeutics to inhibit the PTPN2-related catalytic function represents an attractive approach. Except for two non-competitive inhibitors, all published inhibitors of PTPN22 interact with its active site and exhibit competitive inhibition (85). Compound 8b, with an IC_50_ of 0.26 ± 0.01 µM and at least 9-fold selectivity against various PTPs (86), attenuated early TCR signaling and increased ZAP70 phosphorylation in Jurkat T cells. In mouse thymocytes, the activity of compound 8b was similar to that in the human T-cell line, while *in vivo* it downregulated mast cell action and anaphylaxis (87). Non-competitive PTPN22 inhibitors, such as 4e, have been discovered from a screen of 4,000 drug-like molecules (88). Other inhibitors were identified through various methods, including virtual screening and screening of Au(I) complexes. L-1 (**Table 4C**), a quinolone carboxylic acid scaffold, is the only PTPN22 inhibitor with published *in vivo* pharmacokinetics and reported potential in immunotherapy (80). The L-1 compound was discovered by screening a small collection of drug-like compounds for PTPN22 inhibition (80). L-1 consists of a quinolone derivative core, an L-alanine linker, and a biphenyl carboxylic group, and it competitively inhibits PTPN22 with 7-10 times greater selectivity over other PTPs. Administration of L-1 to mice bearing MC38 or CT26 tumors substantially suppresses tumor growth, particularly when combined with anti-PD-L1, and augments the presence of tumor-associated macrophages as well as CD8^+^ and CD4^+^ T cells. Notably, L-1 treatment does not affect MC38 tumor growth in PTPN22-deficient mice, implying that its therapeutic benefits are mediated through host-expressed PTPN22. The anti-tumor effects of L-1 are partially diminished when tumor F4/80^+^ macrophages are depleted in L-1-treated mice, suggesting that macrophages play a role in L-1’s anti-tumor activity (80). All compounds mentioned above are preclinical and currently there are no PTPN22 inhibitors tested in the clinic.

### SHP-1

The tyrosine phosphatase SHP-1 (PTPN6) is expressed in mature hematopoietic lineages and, in a different isoform, in endothelial cells (89). It has 95% homology between humans and mice, making it a suitable target for preclinical mouse studies (90). SHP-1 is composed of three domains: N-terminal SH2, C-terminal SH2, and catalytic PTP domains. The N-terminal SH2 domain is auto-inhibitory until the C-terminal SH2 domain binds to a phosphopeptide ligand; maximal phosphatase activity occurs when both SH2 domains are engaged (91). At intracellular level, SHP-1 likely interacts with inhibitory-receptor superfamily proteins containing immunoreceptor tyrosine-based inhibitory motifs (ITIMs) (92), such as leukocyte associated immunoglobulin like receptor 1 (LAIR-1), PD-1, and CTLA-4 (93). Other SHP-1 binding partners and substrates in T cells are not well understood and include Zap70, LCK, PI3K, Vav, and TCRζ (94). In addition, the JAK-STAT pathway is regulated by SHP1 (89).

It was hypothesized that SHP-1 inhibition may enhance adoptive T cell-mediated tumor control. Accordingly, increased SHP-1 activity in tumor-infiltrating lymphocytes is linked to a non-lytic phenotype and its deficiency improves immunotherapy (95). SHP-1 deficient T cell efficacy varies depending on the tumor model and has the potential to boost efficacy of PD-1/PD-L1 inhibition (96). Nevertheless, the exact role of SHP-1 in immunotherapy is unclear and its inhibition could cause toxic side effects. Therefore, SHP-1 may be best suited for adoptive cell transfer therapies (95, 96). Tumor-specific *Shp-1* deficient CD8^+^ T cells exhibit enhanced expansion in response to tumor antigens compared to *Shp-1* reconstituted CD8^+^ T cells (97). In addition, adoptive transfer of these cells improves therapeutic outcomes in mice with disseminated leukemia and prevents tumor metastasis in melanoma models (96, 97). *Shp-1* knockdown combined with CPIs impairs the growth of high-affinity and low-affinity antigen-expressing tumors that are non-responsive to checkpoint blockade alone. This suggests that SHP-1 inhibition may be particularly beneficial for tumors expressing low-affinity antigens with limited responsiveness to CPIs (96). Mice with global, inducible *Shp-1* deletion develop splenomegaly and lung inflammation (98). Tumors from SHP-1^-/-^ mice contain higher percentages of activated CD4^+^ and CD8^+^ T cells and increased effector T cell to Treg cell ratios (98). Noteworthy, in line with its inhibitory role in cell proliferation, migration, and invasion, higher SHP-1 mRNA levels can be linked to improved survival in various carcinomas. As a result, SHP1 is considered a tumor suppressor in these tumor types (99).

The catalytic inhibitor of SHP-1, TPI-1 (**Table 4D**), has been explored as a potential anti-tumor immunotherapy. TPI-1 selectively targets SHP-1 over SHP-2 (10-fold selectivity) and increases phosphorylation of numerous SHP-1 targets (100). TPI-1 treatment enhances the number of IFNγ^+^ cells in mouse and human immune cells and slows tumor growth in immunocompetent mice, but not in athymic nude mice. TPI-1 treatment reduces T cell exhaustion while increasing activation of CD8^+^ tumor-infiltrating lymphocytes in PD-1/PD-L1 blockade-resistant 344SQ lung tumors (93). SHP-1 inhibition may also enhance NK cell anti-tumor activity. Indeed, while TME hypoxia reduces NK cell cytotoxicity, SHP-1 knockdown or TPI-1 inhibition partially restores hypoxic NK cell cytotoxicity (101). As mentioned above, enhancing SHP-1 activation can be beneficial in certain indications and indeed agonistic small molecules have been developed. For example, the sorafenib analogs SC-43 and SC-40 have shown promising anti-tumor activity in a subcutaneous hepatocellular carcinoma model (102). Nevertheless, further investigations are needed at present to precisely define tumor types that would benefit from SHP-1 activation rather than inhibition.

### SHP-2

The *PTPN11* gene encodes for SHP-2, a PTP phosphatase with two N-terminal SH2 domains, a PTP domain, and a C-terminal tail with regulatory functions. In its inactive state, SHP-2 exhibits an auto-inhibited conformation, with the N-terminal SH2 domain interacting with the PTP domain and preventing access to the catalytic site (54, 103). Activation of SHP-2 occurs when bis-phosphotyrosyl peptides (such as IRS-1) or certain interaction partners bind to its SH2 domains (104, 105). SHP-2 is involved in numerous cell signaling pathways (RAS-ERK, PI3K-AKT, JAK-STAT) and functions downstream of various receptor-tyrosine kinases in the cytoplasm, although its precise role is not yet fully understood (106, 107). Mutations in the *PTPN11* gene that lead to SHP-2 hyper-activation have been identified in Noonan syndrome, juvenile myelomonocytic leukemia, myelodysplastic syndrome, B-cell acute lymphoblastic leukemia, acute myeloid leukemia, and several solid tumors (107–109).

In T cells, SHP-2 is a key mediator of PD-1 inhibitory receptor signaling through direct interaction with the PD-1 cytoplasmic region’s ITIM motif and immunoreceptor tyrosine-based switch motif (ITSM). As PD-1 expression is induced by T cell activation, SHP-2 may initially promote T cell activation upon TCR ligand engagement and later downstream signaling by entering in complex with PD-1. Although multiple studies have reported a PD-1-SHP-2 interaction, the exact nature of the complex and SHP-2’s functional necessity in PD-1 signaling remain unclear (110, 111). Despite the strong evidence for the formation of a PD-1-SHP-2 complex, some studies indicate that PD-1 signaling can inhibit T cell activation even without SHP-2. It is possible that in certain cellular contexts SHP-1 compensates for the loss of SHP-2 to maintain PD-1 signaling, or PD-1 can inhibit T cell activation without the presence of either phosphatase (112, 113). In mouse xenograft models, T cell-specific *Shp-2* deletion shows variable results. For instance, T cell SHP-2 deficiency in metastatic melanoma model does not affect survival and late-stage tumor size, but actually increases metastasis (114). *Shp-2* specific deletion in T cells does not affect tumor growth and tumor-infiltrating lymphocytes in the MC38 colon adenocarcinoma model (114). Also, anti-PD-1 antibody treatment improves tumor control regardless of the mouse genotype, suggesting PD-1 inhibitory function can occur without SHP-2. Conversely, another study shows that T cell-specific SHP-2 deletion enhances control of MC38 tumor growth, with tumors exhibiting increased activated CD8^+^ T cells (115). Deletion of SHP-2 in myeloid cells curbs B16 melanoma growth and boosts tumor concentrations of chemoattractant CXCL9, macrophage-produced IFNγ-induced CXCL9, and CD8^+^ T cell infiltration into tumors. Consistently, blocking CXCL9 or IFNγ stimulates tumor growth in mice with myeloid SHP-2 deficiency (116).

Several allosteric SHP-2 inhibitors are being investigated in clinical trials for cancer treatment. These molecules act by stabilizing SHP-2’s auto-inhibited state and show high selectivity over other enzymes. The two most advanced compounds, currently in phase 1 and 1/2 trials, are SHP099 (**Table 4E**) and TNO155 (**Table 4F**) (NCT03114319, NCT04000529, NCT04330664, NCT04294160, NCT04292119, NCT04185883 and NCT04699188). SHP099 has shown potential in mouse tumor models, reducing growth in colon and breast cancers. Its anti-tumor effects depend on immune cells, since SHP099’s efficacy is reduced in immunocompromised mice. Combined with anti-PD-1, SHP099 further inhibits tumor growth. Similarly, orally administered TNO155 suppresses MC38 tumor growth as a single agent and CT26 tumor growth with anti-PD-1 combination. Combined with anti-PD-1, SHP099 or TNO155 enhance CD8^+^ T cell activation and tumor infiltration while reducing overall immune cell infiltration and altering tumor-associated macrophage composition (115, 117). SHP099 administered in combination with radiation and anti-PD-L1 improves control of resistant tumors, enhances survival and reduces metastases (118). It does not affect cell viability *in vitro*, and its benefits mainly rely on anti-tumor immunity, as confirmed by CD8^-/-^ or F4/80-expressing cell depletion. SHP-2 inhibition combined with KRAS-G12C inhibitors enhances immunity against KRAS^G12C^ mutated tumors. SHP099 improves KRAS-G12C inhibitor efficacy and, with ARS1620, increases survival and tumor regression in mouse models. Combined with anti-PD-1, SHP099–ARS1620 is more effective than the single agents, boosting ARS1620 efficacy in patient-derived cancers and altering immune cell populations (119).

RMC-4550 (WO2020/247643) is an allosteric SHP-2 inhibitor developed by Revolution Medicines. Its derivative RMC-4630 (**Table 4G**) is in clinical trials with early results indicating disease control in five of seven patients with KRAS^G12C^ NSCLC (120). Following oral administration, RMC-4550 decelerates tumor growth in mouse models through a T cell-mediated effect. It enhances inhibition with anti-PD-1, anti-CTLA4, or anti-CSF1R antibodies and, combined with anti-PD-1, extends the time to reach tumor burden. RMC-4550 modifies immune cell composition and boosts MHC-I and PD-L1 expression. It does not impact T cell proliferation or cytokine release, but counteracts myeloid-derived suppressor cells (MDSCs)’ suppressive effects on CD8^+^ T cells (121). The SHP-2 inhibitor IACS-13909 was developed through collaboration between Navire Pharma and MD Anderson Cancer Center (65). Its derivative, BBP-398 (**Table 5H**), is in phase 1 trials as monotherapy and with PD-1 and KRAS^G12C^ inhibitors. Jacobio’s JAB-3068 (**Table 4I**) and JAB-3312 (**Table 4J**) are in phase 1/2 trials as monotherapy and combined with PD-1 or MEK inhibitors. Relay Therapeutics’ RLY-1971 (**Table 4K**) is undergoing phase 1 trials as monotherapy and with a KRAS^G12C^ inhibitor.

**Table 5:** PGE2 receptor EP2/EP4 antagonists.

Compound	Target	Indication/Combination Preclinical model	Phase	Company	Reference
Grapiprant (ARY-007/IK-007)	EP4	Solid tumors (+ pembrolizumab) (+ eribulin mesylate)	1/2	Arrys Therapeutics Ikena Oncology	NCT03696212 NCT05041101
ONO-4578 BMS-986310	EP4	Advanced solid tumors (+ nivolumab)	1/2	Bristol-Myers Squibb Co	NCT03661632 NCT03155061
cr-6086	EP4	Metastatic colorectal cancer (+ balstilimab)	1b/2a	Rottapharm Biotech	NCT05205330
INV-1120	EP4	Advanced solid tumors (+ pembrolizumab)	1a/1b	Shenzhen Ionova Life Science Co Ltd	NCT04443088
Palupiprant (AN-0025)	EP4	Advanced solid tumors, metastasis (+ pembrolizumab)	1	Adlai Nortye Pharmaceutical Co Ltd	NCT04432857
DT-9081	EP4	Advanced solid tumors	1	Domain Therapeutics SA	NCT05582850
KF-0210	EP4	Advanced solid tumors (+ atezolizumab)	1	Keythera Pharmaceuticals Co Ltd	NCT04713891
TPST-1495 (A)	EP2+EP4	Advanced solid tumors (+ pembrolizumab)	1	Tempest Therapeutics Inc	NCT04344795 WO2019/204523
MBF-362 (B)	EP2+EP4	Advanced solid tumor	1	Medibiofarma	NCT05940571 WO2020/161275
ACT-1002-4391 (C)	EP2+EP4	Mouse breast cancer model	Preclinical	Idorsia Pharmaceuticals Ltd.	WO2018/210994 Jeay S, 2023 #34
KNP-502 OCT-598 (D)	EP2+EP4	Mouse lung/colorectal/breast cancer model	Preclinical	Kanaph Therapeutics	WO2022/039563 (122)
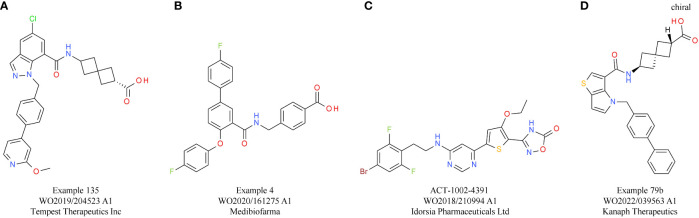					

Considering its significant function, SHP-2 serves as a potential target for cancer treatment, and the scientific community has already put considerable effort into the development of SHP-2 inhibitors, particularly allosteric inhibitors. Despite significant advancements on both preclinical and clinical levels in recent years, the potent and selective SHP-2 inhibitors that have been reported are still in the early stages of clinical trials. Nevertheless, the discovery of additional biological roles of SHP-2 through the application of these compounds in various contexts will facilitate their applicability in treating other diseases as well.

## Small molecules inhibiting immunosuppressive components of the TME

### PGE2 receptors EP2/EP4

**Figure 2:**
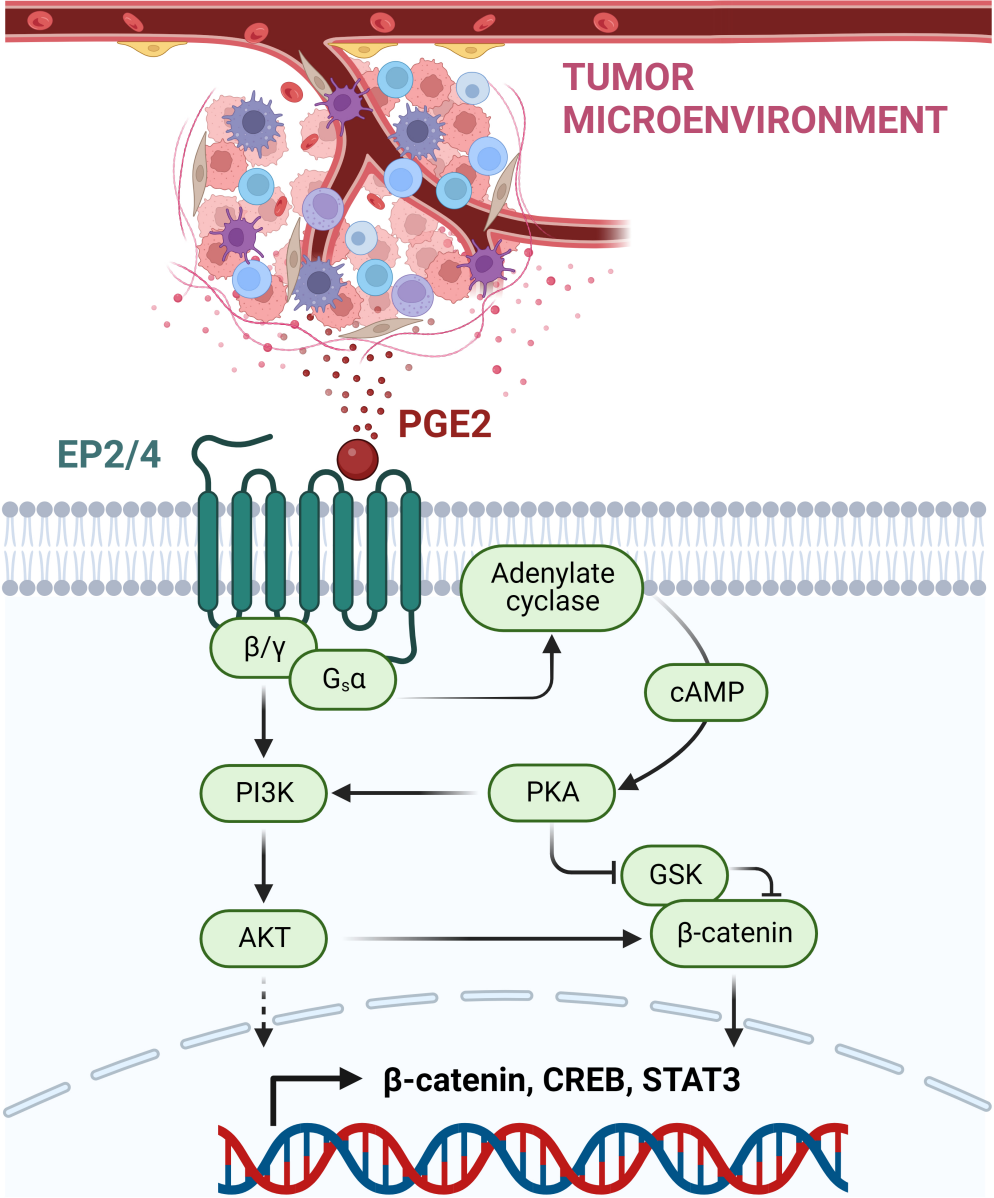
PGE2-mediated immunosuppressive, pro-tumorigenic signaling via EP2/EP4 receptors. PGE2 is released within the TME by various immune cells and cancer cells. By binding to the GPCRs EP2 and EP4, PGE2 mediates the induction of the coupled G-protein subunits G_s_α and β/γ. This allows the activation of the PI3K-AKT pathway and the induction of the adenylate cyclase with subsequent cAMP release. High levels of cAMP mediate PKA kinase activation, which then can inhibit GSK, acting as a negative regulator of β-catenin. Next to β-catenin, other transcription factors are induced, including CREB and STAT3, resulting in the suppression of anti-tumor immune response and promotion of cancer development and progression. This figure was created with BioRender.com.

PGE2 is a bioactive lipid synthesized by cyclooxygenase (COX) enzymes. This prostaglandin acts on four G-protein-coupled receptors (GPCR): prostaglandin E receptor (EP)1-EP4. These receptors are expressed on a multitude of cell types, where they regulate diverse physiological functions, including a wide range of immune response processes (123). Besides different tissue expression patterns, the EP receptors also differ in their PGE2 binding affinity: EP3 and EP4 were shown to be high-affinity receptors, whereas EP1 and EP2 require higher concentrations of PGE2 for signaling induction (124). As GPCRs, these receptors induce intracellular signaling events via their coupled G-proteins. EP1 is coupled to a Gαq subunit, which mediates the induction of the nuclear factor of activated T cells (NFAT), NFκB and the MAPK pathway via activation of PKC. In contrast, EP2 and EP4 receptors are both coupled to Gαs proteins, inducing the activation of the protein kinase A (PKA)/cAMP-responsive element binding protein (CREB) as well as the glycogen synthase kinase 3 (GSK3)/β-catenin pathway (**Figure 2**). The EP3 receptor occurs in multiple splice variants, which allows interaction with different G-proteins including Gi, Gs, and G13. The most abundant EP3 splice variant interacts most likely with a Gi protein, leading to the activation of adenylate cyclase and activation of the MAPK and RAS/RAF signaling cascade (124, 125).

In the context of tumor development, the COX-2/PGE2 axis has been shown to be upregulated in a wide set of tumor tissues, contributing to multiple processes ranging from growth promotion and tumor initiation to angiogenesis and metastasis (125, 126). Roles in cancer development have been described for all PGE2 receptors (EP1-EP4) (127). In the TME, elevated PGE2 acts as an immunosuppressive agent, dampening anti-tumor immunity and promoting tumor immune escape and cancer progression (125). This negative regulation of the anti-tumor immune response is predominantly carried out via EP2 and EP4 receptor signaling. In addition to their presence on tumor cells, both receptors are expressed on different sets of immune cells, including MDSC, DCs, macrophages, NK cells, and Tregs. Together these cell types set the immunosuppressive environment of the TME (125, 126). MDSCs act in the TME through the secretion of different molecules, including nitric oxide synthase, reactive oxygen species, arginase as well as TGFβ and IL10, which mediate M2 macrophage polarization, suppression of NK cell activities and induction of Tregs. In addition, MDSCs are able to dampen effector CD8^+^ T lymphocytes (126, 128, 129). MDSC differentiation was shown to be induced by PGE via EP4 and/or EP1/2 (130). In macrophages and DCs, PGE2 supports the polarization towards the immunosuppressive M2-like subtype and dampens DC differentiation, an effect mainly mediated through the EP4 receptor as demonstrated by EP4 receptor deletion and antagonist studies (131–134). Conventional DC1s (cDC1s) are required in the TME to present tumor-specific antigens via their MHC class I complex to CD8^+^ T cells (135–137). CDC1 recruitment to the tumor side is critically mediated through chemokine secretion by NK cells, which is another process counteracted by PGE2 produced by the tumor tissue. PGE2 was not only shown to negatively affect NK viability and chemokine secretion, but also chemokine receptor expression in cDC1 cells themselves (138). In addition, NK cell cytotoxicity was shown to be decreased by PGE2 via the EP2 and EP4 receptors (139). Moreover, EP2 and EP4 regulate the PGE2-mediated activation of immunosuppressive Tregs (140). For cytotoxic CD8^+^ T cells targeting antigen-presenting tumor cells, tumor infiltration was shown to be regulated via EP4 as well (131).

Based on its extensive role as an immune mediator in the context of cancer development, efforts to target PGE2 as part of cancer immunotherapy are underway. The evident approach of preventing PGE2 synthesis has been addressed using non-steroidal anti-inflammatory drugs and COX-2 inhibitors such as celecoxib. However, despite demonstrated clinical efficacy, these drugs cause severe adverse events due to their broad inhibition of prostanoid production (141). Therefore, the antagonism of PGE2 receptors, especially EP2 and EP4 based on their role in the TME, has become an attractive approach in cancer therapy. Only preliminary *in vitro* data are available for targeting EP2, such as for the tool compound PF-04418948 from Pfizer (142). In contrast, multiple selective EP4 inhibitors are already in clinical trials as single agent therapy or in combination with CPIs (See **Table 5**).

Preclinical *in vivo* studies showed promising results in mouse models, including breast cancer and colon cancer models (143–145). In addition to single receptor targeting, also the concept of a dual inhibition of EP2 and EP4 is under evaluation. The most advanced candidate is TPST-1495 (**Table 5A**) from Tempest Therapeutics, with encouraging preclinical *in vivo* data showing reduction of colorectal tumorigenesis and prolonged overall survival. In this study, the dual EP2/EP4 antagonist did not only demonstrate clear superiority compared with single EP2 or EP4 antagonists and celecoxib treatment, but also a slightly higher efficacy than an EP2/EP4 inhibitor combination treatment. Importantly, CPI synergy was also shown using an anti-PD-1 antibody (146). Currently, TPST-1495 is under evaluation in a first-in-human clinical phase 1 study, with initial results showing a manageable safety profile and stable disease up to partial remission effects for patients with certain tumor subtypes (147). MBF-362 (**Table 5B**) from Medibiofarma also entered clinical phase 1 evaluation recently (NCT05940571). In addition, other dual EP2/EP4 antagonists, such as ACT-1002-4391, have already shown promising preclinical results for solid tumor therapy (148) (**Table 5C**).

Further clinical data are needed to assess whether dual EP2/EP4 antagonists can tackle the extensive immune suppressive effects mediated by PGE2 within the TME, thereby restoring the anti-tumor immunity of the host and opening the possibility to overcome resistance to CPIs due to a non-inflamed tumor environment.

## Negative regulators of the cGAS-STING pathway

**Figure 3:**
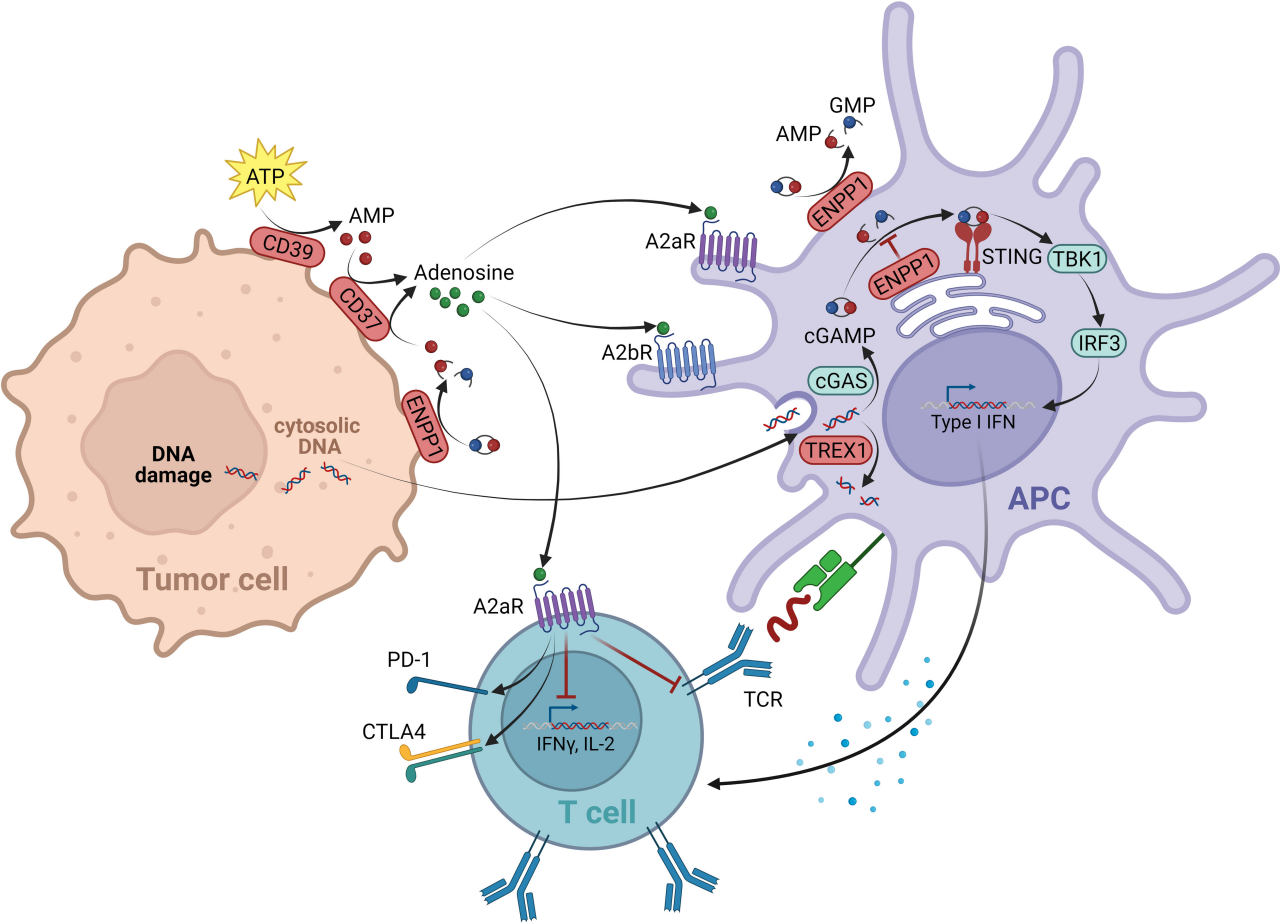
Negative regulators within the cGAS/STING and adenosine signal network. The cGAS-STING cascade is able to sense cytosolic DNA fragments that can originate from tumor cells. The enzyme cGMP-AMP synthase (cGAS) produces the second messenger cGAMP upon DNA binding, which binds and activates STING located at the ER. STING then translocates to perinuclear regions, where it binds and activates the kinase TBK1. The TBK1-STING complex is subsequently able to bind the transcription factor IRF3, which is being phosphorylated by TBK1. IFN3 then dissociates from the complex and induces the expression of several target genes, including IFNβ. As negative regulators of the cGAS-STING pathway, on one side the exonuclease TREX1 is in place, degrading cytosolic DNA, thereby reducing the ligand of cGAS. On the other hand, the ectonucleotide pyrophosphatase/phosphodiesterase 1 (ENPP1) mediates the degradation of the STING ligand cGAMP to AMP. With AMP being the source of adenosine production, ENPP1 contributes to the immunosuppressive adenosine pathway. Adenosine is elevated in the TME. It is generated from AMP via ectonucleotidase CD73. Next to the AMP source from ENPP1, the main source of AMP for adenosine production originates from the ATP breakdown mediated by CD39. The immunosuppressive effect of eADO is mediated via GPCRs A2aR (expressed on all immune cells) and A2bR (expression restricted to myeloid cells). On T lymphocytes A2aR mediates a suppression of TCR signaling resulting in an overall negative regulation of T cell activation, proliferation and survival. On DCs mainly A2bR signaling alters the expression of various immunomodulating factors, resulting in immunosuppressive downstream signaling events. This figure was created with BioRender.com.

The STING pathway belongs to the innate immune system, responsible for the recognition of endogenous “danger associated molecular patterns” (DAMPs). Via the cytosolic enzyme cGMP-AMP synthase (cGAS), the cGAS-STING cascade senses cytosolic DNA fragments released either from pathogens or occurring endogenously due to cellular damage and stress. Upon DNA binding, cGAS produces the second messenger cGAMP, a cyclic dinucleotide. CGAMP binds to STING, which resides as a transmembrane protein at the ER, leading to its activation. STING then translocates via the Golgi apparatus to perinuclear regions. In this process, the TANK-binding kinase 1 (TBK1) interacts with STING, resulting in STING phosphorylation and TBK1 auto-phosphorylation. The STING-TBK1 complex is ultimately bound by the interferon regulatory transcription factor (IRF3), which upon its phosphorylation by TBK1, dissociates from the complex to induce the expression of several target genes, most importantly IFNβ (149) (**Figure 3**).

Several negative regulatory control mechanisms are in place to avoid an over-activation of cGAS-STING signaling, which would lead to an excessive, disproportional immune response. As such, cGAS-STING induces autophagy, most likely to reduce cytosolic DNA levels, which are a stimulus of the pathway (150, 151). Furthermore, active STING-TBK1 complexes are targeted by lysosomal degradation, reducing downstream signaling (152, 153). In addition, the pathway is controlled by specialized enzymes acting as negative regulators: the ectonucleotide pyrophosphatase/phosphodiesterase 1 (ENPP1) mediates the degradation of the STING ligand cGAMP (154, 155); and the levels of tumor-derived DNA, as the upstream stimulus of cGAS, are negatively controlled through the three-prime exonuclease 1 (TREX1) by cytosolic DNA degradation (156) (**Figure 3**).

In the context of CIT, the cGAS-STING signaling cascade is critical for sensing of immunogenic tumors. Apart from tumor-intrinsic cGAS-STING induction that could contribute to a beneficial therapeutic outcome (157, 158), a critical event here is the production and release of cGAS-STING agonists that stimulate immune cells. It is thought that tumor cells either transfer cGAMP itself or tumor-derived DNA to antigen-presenting cells within the TME, which subsequently initiate the adaptive immune T cell response (159, 160). The importance of this process for anti-tumor immunity was shown in preclinical *in vivo* models, with STING^-/-^ mice showing defective tumor control. In line with this, deficient CD8^+^ T cell priming and anti-tumor T cell accumulation (with IFNβ being the crucial link between the innate and the adaptive immune response) was reported (159, 161). Studies with intratumoral administration of STING agonists further demonstrated profound tumor regression and substantial systemic immune responses, emphasizing cGAS-STING induction as an attractive therapeutic strategy for cancer (162).

Several generations of cGAS-STING agonists have already been developed. However, their application is often restricted to intratumoral injections and a key limitation is the risk of an overstimulation leading to severe side effects such as cytokine storms and T cell death (149, 163, 164). Therefore, alternative approaches of cGAS-STING pathway induction for cancer immunotherapy are being sought.

An alternative strategy that came into focus is the indirect activation of the cGAS-STING pathway via inhibition of its negative regulators, allowing systemic administration of small molecules. One of the targets evaluated is ENPP1, responsible for cGAMP and ATP degradation, a process that also leads to the generation of the immunosuppressant AMP. Therefore, ENPP1 inhibition not only increases cGAMP levels for STING activation, but also reduces AMP levels. The induction of the immunosuppressive adenosine pathway is discussed in more detail below (155) (**Figure 3**). A relevant role of ENPP1 in tumor development could already be seen in mouse *in vivo* studies. For example, in a breast cancer model, ENPP1 overexpression led to enhanced tumor metastasis in the bone (165). Several small molecule ENPP1 inhibitor candidates are currently in preclinical investigations, with encouraging initial results being disclosed recently. The leading candidate from Stingray Therapeutics SR-8541A (**Table 6B**) showed decreased tumor growth in a CT26 colon cancer model. Radiotherapy synergy and abscopal anti-tumor response in another colorectal carcinoma mouse model (MC38) was also reported (166). ENPP1 inhibitors from Mavupharma (MV-626) (**Table 6C**) demonstrated monotherapy activity and synergy with PD-L1 treatment in the same mouse model (167). For another preclinical candidate from Avammune Therapeutics (AVA-NP-695) (**Table 6D**), synergies with radiotherapy and DNA damage response inhibitors were shown in a breast cancer model (168). The first candidate in a clinical phase 1a/b study is RBS-2418 from Riboscience LLC (**Table 6A**). It is clinically assessed as monotherapy or in combination with pembrolizumab in patients with advanced unresectable, recurrent, or metastatic tumors (NCT05270213, NCT05683470). Initial results showed good oral bioavailability and tolerability as well as no dose-limiting toxicity for both the monotherapy and the combination. Furthermore, effects on immune biomarkers were observed, including modulation of cDCs levels and T cell proliferation and activation (169).

**Table 6:** ENPP1 and TREX antagonists.

Compound	Target	Indication/Combination Preclinical model	Phase	Company	Reference
RBS-2418 (A)	ENPP1	Adrenal gland tumor; Advanced solid tumor; Metastasis (+ pembrolizumab)	1	Riboscience LLC	NCT05270213 NCT05683470 WO2022/197734
SR-8541A (B)	ENPP1	Mouse breast tumor, colon tumor model	Preclinical	Stingray Therapeutics	WO2021/158829 (166)
MV-626 (C)	ENPP1	Mouse colon tumor, pancreatic ductal adenocarcinoma model	Preclinical	AbbVie Inc Mavupharma	WO2019/191504 (167)
AVA-NP-695 (D)	ENPP1	Mouse breast cancer model	Early preclinical	Avammune Therapeutics	WO2021/053507 (168)
CPI-38 (Example E)	TREX1	Mouse colorectal tumor model	Preclinical	Constellation Pharmaceuticals	WO2021/016317
Compound 4A (F)	TREX1	Mouse colorectal tumor model	Preclinical	Tempest Therapeutics, Inc.	WO2021/263079
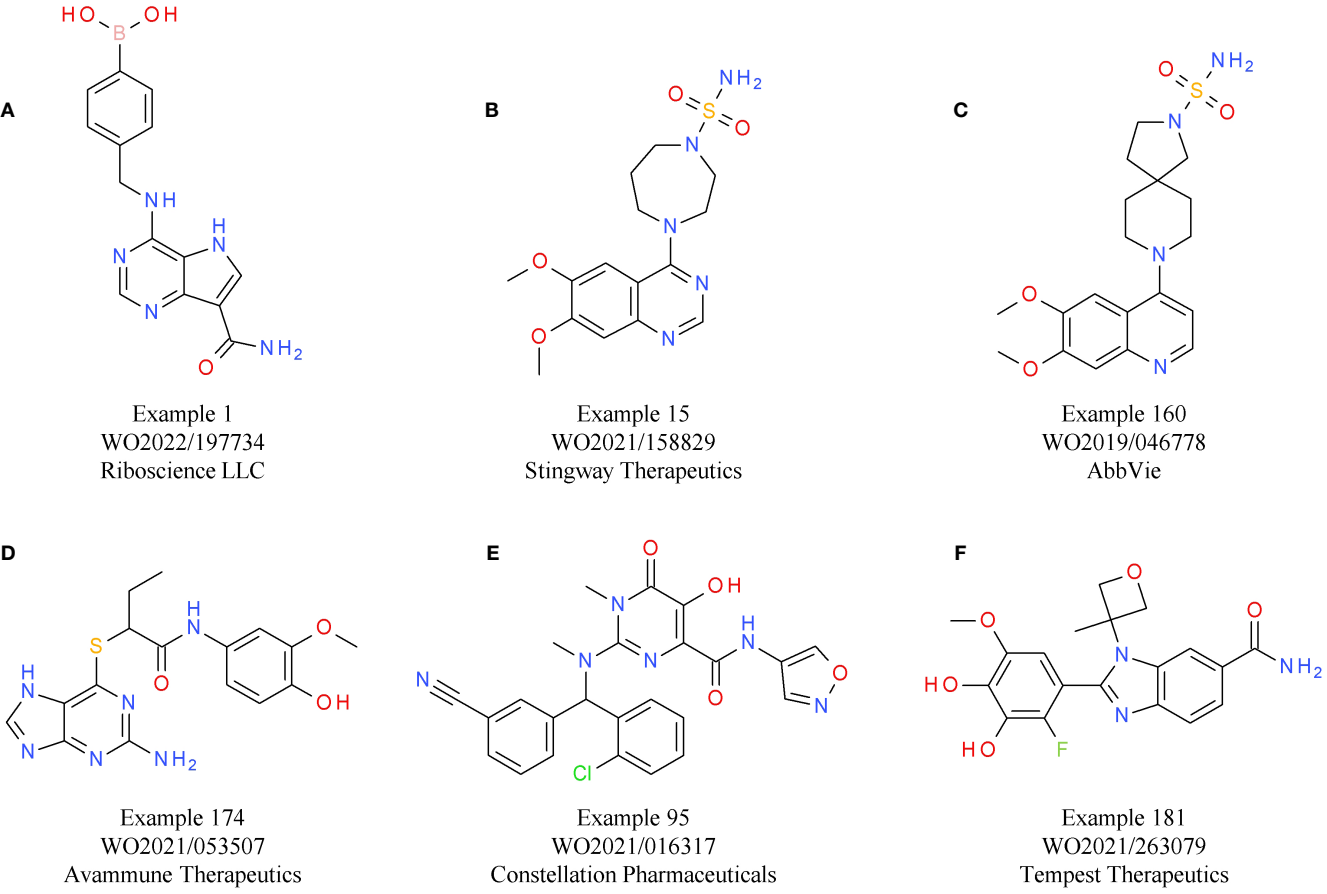					

Another gatekeeper enzyme of the cGAS-STING pathway that has been linked to the TME and cancer development is the exonuclease TREX1 (156). TREX1 counteracts potential cGAS-STING induction in the TME and thereby the anti-tumor immune response by degrading tumor-derived DNA that is either spontaneously generated or induced by DNA damaging therapeutic approaches (156). Accordingly, TREX1 activity was shown to be negatively correlated with outcomes in multiple cancers and its upregulation is associated with cancer cell treatment, in particular radiotherapy (170, 171).

Similar to inhibition of ENPP1, targeting TREX1 with small molecules is an attractive therapeutic strategy to enhance the cGAS-STING axis without using direct cGAS-STING agonists. Currently, several candidates are in preclinical development. The TREX1-targeting small molecule CPI-38 from Constellation (**Table 6E**) demonstrates decreased tumor growth in the MC38 (colorectal carcinoma) syngeneic tumor model, both as a standalone treatment and when combined with anti-PD-1 (172). In addition, an orally available TREX1 inhibitor (**Table 6F**) developed by Tempest Therapeutics shows anti-tumor activity in combination with DNA-damaging agents in the colon carcinoma tumor model CT26 (173). Further programs are run by SpringBioscience AB.

## Negative regulators of the adenosine pathway

The metabolite adenosine is another key mediator within the TME that contributes to an immunosuppressive and pro-tumorigenic environment. In the extracellular space of healthy tissue, adenosine levels are relatively low. However, in tumor tissue high concentrations of extracellular adenosine (eADO) are observed. As a breakdown product of pro-inflammatory ATP, secreted in response to cellular stresses such as tissue injury, ischemia, or cell death, eADO supports the negative feedback loop following and counteracting inflammatory immune responses. In the context of tumor development, these immune-dampening processes mediated by eADO favor tumor immune escape (174, 175). The ectonucleotidase CD39 (ectonucleoside triphosphate diphosphohydrolase 1) hydrolyzes ATP to ADP and AMP. AMP is subsequently converted to eADO through a second ectonucleotidase, CD73 (5′-nucleotidase) (176). Despite CD39 and CD73 being considered the major sources of eADO, other enzymes contribute to eADO production, including CD38 (ADP-ribosyl cyclase/cyclic ADP-ribose hydrolase 1) and ENPP1 (177) (**Figure 3**).

The immunosuppressive effect of eADO is mediated via the adenosine receptors A2aR and A2bR, both acting as GPCRs. A2aR is expressed by nearly all immune cell types and binds eADO with high affinity. In contrast, the expression of A2bR is limited mainly to myeloid cells and, because of its low affinity properties, its activity becomes relevant mostly in pathophysiological situations with elevated eADO levels (178, 179). On T lymphocytes, A2aR mediates suppression of TCR signaling itself, next to impaired CD28 costimulatory, as well as IL2R signaling. A2aR signaling ultimately results in an overall negative regulation of T cell activation, proliferation, and survival. In addition, the upregulation of co-inhibitory receptors was reported, including PD-1 and CTLA-4 (174) (**Figure 3**). Furthermore, A2aR suppresses the cytotoxic function of NK cells and impairs antigen receptor signaling of B cells (180, 181). On myeloid cells, A2aR and A2bR can promote differentiation of macrophages towards the pro-tumorigenic “M2-like” phenotype (182). Similarly, in DCs mainly A2bR signaling alters the expression of various immunomodulating factors, including cytokines such as IL10 or TGFβ or enzymes such as COX2, all leading to immunosuppressive downstream signaling events (183).

Based on the comprehensive immunosuppressive effects of eADO, eADO-mediated pathways are being investigated for various CIT approaches. Efforts are focused either on the inhibition of the eADO receptors A2aR and A2bR or on the prevention of eADO production via inhibition of CD39 and CD37. Regarding eADO receptors, multiple selective small molecule inhibitors are under clinical investigation, including ciforadenant (CPI-444), taminadenant (NIR178), imaradenant (AZD4635), etrumadenant (AB928), and inupadenant (EOS-850) (184) (**Tables 7A–C**). Results from early phase trials showed a modest but consistent overall response rate (ORR) around 5% for the monotherapy, which increased up to 15% upon combination with CPIs (175). In a phase 1 trial in treatment-refractory renal cell cancer (RCC), ciforadenant (Corvus Pharmaceuticals) achieved an ORR of 3% as monotherapy and 11% in combination with atezolizumab, a PD-L1 inhibitor (185). To achieve a potential increase in efficacy, the development and characterization of dual A2aR and A2bR inhibitors is also pursued. One dual inhibitor candidate is etrumadenant from Arcus, which is currently tested in four clinical phase 2 trials as monotherapy as well as in combination with immunotherapies. In the preceding phase 1 study, the combination of etrumadenant, zimberelimab (anti-PD-1 antibody), and docetaxel showed a manageable safely profile and clinical benefit in patients with metastatic castrate-resistant prostate cancer (mCRPC) (186).

**Table 7:** A2aR, A2bR and CD73 antagonists.

Compound	Target	Indication/Combination Preclinical model	Phase	Company	Reference
Ciforadenant (CPI-444) (A)	A2aR	NSCLC/ RCC/TNBR*/ malignant melanoma (+ ipilimumab, nivolumab) (+ atezolizumab)	1b/2	Corvus Pharmaceuticals	NCT05501054 NCT02655822
Taminadenant (NIR178) (B)	A2aR	Advanced solid tumor/NSCLC (+ spartalizumab, taminadenant)	1-2	Novartis	NCT03207867
Imaradenant (AZD4635)	A2aR	Advanced solid tumor/mCRPC*/NSCLC (+ abiraterone, docetaxel, durvalumab, enzalutamide, imaradenant, oleclumab, prednisone)	1-2	AstraZeneca	NCT02740985
Inupadenant (EOS-850)	A2aR	Metastatic solid tumors i.a. NSCLC/colon/breast/bladder cancer (+carboplatin, inupadenant, pemetrexed disodium) (+pembrolizumab)	1-2	iTeos Therapeutics SA	NCT05403385 NCT03873883
Etrumadenant (AB928) (C)	A2aR A2bR	Lung/prostate/colorectal cancer (+ domvanalimab, zimberelimab) (+ docetaxel, enzalutamide, quemliclustat, zimberelimab, sacituzumab govitecan) (+ bevacizumab, m-FOLFOX-6, regorafenib)	1-2	Arcus Biosciences Inc	NCT04262856 NCT04381832 NCT05633667 NCT04660812
TT-702 (D)	A2bR	Advanced solid tumor i.a. mCRPC*/TNBC*/MSI/MMR*	1/2	Teon Therapeutics	NCT05272709 WO2022/256550
Quemliclustat (AB680) (E)	CD73	Lung/gastrointestinal/pancreatic cancer (+ domvanalimab, zimberelimab, docetaxel, platinum-based doublet) (+ fluorouracil, leucovorin, oxaliplatin) (+ nab-paclitaxel, gemcitabine)	1b/2	Arcus Biosciences Inc	NCT05676931 NCT05329766 NCT04104672
ORIC-533 (F)	CD73	Multiple myeloma	1	ORIC Pharmaceuticals Inc	NCT05227144 WO2021/087136
ATG-037 (G)	CD73	Advanced solid tumor (+ pembrolizumab)	1b	Calithera Biosciences Inc/Antengene Corporation Limited	NCT05205109 WO2020/257429
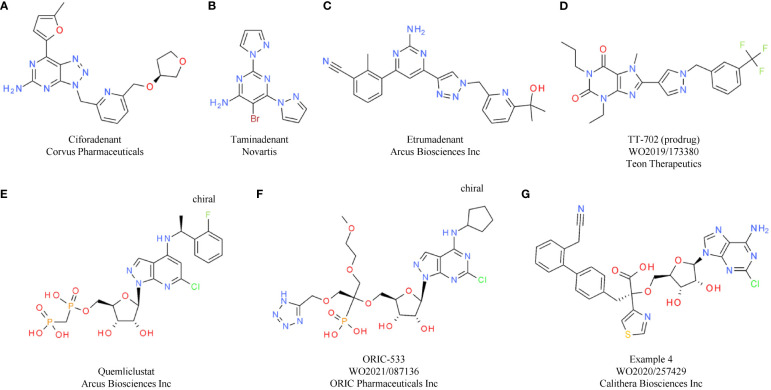					

*TNBC, triple negative breast cancer.

*mCRPC, metastatic castrate resistant prostate cancer.

*MMR/MSI, mismatch repair/microsatellite instability defective tumors.

TT-702 from Teon Therapeutic (**Table 7D**) is a first-in-class selective A2bR inhibitor in development, aiming to selectively inhibit the main receptor type induced by pathologically elevated eADO levels within the TME. This compound showed promising preclinical single agent and synergistic inhibitory effects in combination with anti-PD-1 in CT26 and B16F10 mouse models and is currently under evaluation in a first clinical phase 1/2 study (187).

Inhibiting eADO production by targeting the CD39-CD73 axis is also currently being investigated in the clinic with several monoclonal antibody therapies (175) as well as small molecule inhibitors targeting the two receptors. Three leading candidates targeting CD73 are under evaluation: Quemliclustat (Arcus Biosciences Inc), ORIC-533 (ORIC Pharmaceuticals Inc), and ATG-037 (Antengene Corporation Ltd). Quemliclustat (**Table 7E**) already showed promising results in a phase 1/1b study as a combination with standard of care chemotherapy and zimberelimab (anti-PD-1), with an ORR of 41% in patients with pancreatic cancer (188). Follow-up combination studies for lung and gastrointestinal cancer are ongoing. ORIC-533 from ORIC Pharmaceuticals Inc (**Table 7F**) is evaluated in multiple myeloma in a phase 1 study. Multiple myeloma is a highly relevant cancer type for CD73 inhibition, as it is adenosine enriched. Consistent with this, high CD73 and adenosine levels are associated with poor prognosis and therapeutic resistance (189). The third clinical CD73 inhibitor, ATG-037 from Antengene Corporation Ltd (**Table 7G**), is currently being tested in a phase 1b study as monotherapy and in combination with CPIs. Preclinically, this molecule already demonstrated more potent CD73 enzyme inhibition compared with anti-CD73 antibodies in the clinic (190). Similar to CD73, multiple inhibitory antibodies targeting CD39 have reached the clinic. However, the development of small molecule inhibitors targeting CD39 is still in the discovery phase. With a high activity in this field and several candidates under evaluation in later stage combinational clinical trials, the impact of adenosine pathway inhibition for advancing CIT for different cancer types will become clearer in the near future.

## Biomarker landscape for small molecule negative regulators in cancer immunotherapy

In recent years, CPIs targeting PD-1, PD-L1, or CTLA-4 have shown long-lasting efficacy in cancer treatment. Either as single agents or as part of combination regimens, CPIs have become standard of care for an increasing number of solid tumors. Recent clinical trials in metastatic settings reveal improved response and survival rates when CPIs are combined with other treatments such as chemotherapy and antiangiogenics (191).

Nevertheless, many patients do not benefit or do not have sustained responses to CPIs. The variable CPI responses across patients and cancers is poorly understood. Thus, the identification of novel biomarkers is crucial to improve therapeutic outcomes (192). Currently, predictive biomarkers for CPIs are focused on PD-L1 expression and the immunogenicity of the respective tumor type. The recognition of a tumor by the immune system is largely dependent on a functional antigen presentation machinery and on a high level of tumor neoantigen being presented. The latter is frequently referred to as the “tumor mutational burden”.

Hence, other biomarkers, potentially derived from tissue, blood, microbiota, and tumors, are being sought. In the following section, we provide an overview of the current status of target-specific biomarkers (monitoring and predictive biomarkers), which could be, or are already, used at clinical level to improve therapeutic outcomes.

### MAP4K1

With respect to MAP4K1, the most obvious clinical monitoring biomarker is the direct phosphorylation of the substrate SLP76. TCR re-stimulation is needed to measure changes of pSLP76 in T cells because small molecule-dependent MAP4K1 inhibition is only detectable when TCR signaling gets activated. Apart from this technical detail, monitoring substrate phosphorylation is an approach that will most likely be followed in clinical trials.

Data from the TWT-101 clinical study presented by Treadwell Therapeutics showed CFI-402411 exposures over several days at different treatment time points, with steady-state exposure achieved after approximately 3-6 days (18). Since pharmacodynamic data from patient samples were not available, the pharmacodynamics were modeled based on *in vitro* pSLP76 IC_50_ values, which indicated that the biomarker pSLP76 was fully inhibited at C_trough_ with doses of 560 mg/kg/day (18). Nimbus Therapeutics has not presented clinical pharmacodynamic data, but preclinical experiments imply that pSLP76 was used for measuring target engagement *in vivo*, with the aim of linking pSLP76 modulation with inhibition of tumor growth (193). Accordingly, it can be speculated that monitoring treatment-induced changes in the clinic will rely on pSLP76 as a biomarker as well.

To directly assess immunomodulation induced by treatment with a MAP4K1 inhibitor, Pfizer is analyzing changes of intratumoral T cells using paired biopsies pre- and post-treatment (NCT05233436).

### DGKα/ζ

In the case of DGKα/ζ, it is challenging to measure kinase activity from patient samples because quantification of lipid phosphorylation is technically difficult. Since direct substrate phosphorylation cannot be assessed from clinical specimens, the approach pursued is to analyze enhanced T cell responses in the clinic. Bayer has shared details of an assay that measures ERK1/2 phosphorylation in T cells (194). Phosphorylation of ERK1/2 is induced when T cells in whole blood are re-stimulated with anti-CD3/CD28 antibodies and this phosphorylation is dose-dependently increased by pre-treatment with the DGKζ inhibitor BAY-2965501. This assay is used for the ongoing clinical trial, but to date no clinical data have been shared (194).

### CBL-B

Assessing inhibition of CBL-B in clinical trials is challenging because ubiquitinations are difficult to track in a cellular context. Hence, Nurix invested in the discovery of biomarkers monitoring drug-dependent modulation of cellular signaling events. Aiming to identify phosphorylation events modulated by CBL-B inhibition, isolated human peripheral blood mononuclear cells were stimulated in the presence or absence of the CBL-B inhibitor NX-1607. A phospho-protein flow assay identified increased phosphorylation levels for PLCγ, ZAP70, and HS1. With this set of proximal biomarkers, Nurix established an assay to measure pHS1 in T cells and determined *in vitro*-/*in vivo*-efficacy correlations in a murine syngeneic efficacy model. Based on data for the first four dose levels tested in the clinic, pHS1 increases in a dose dependent manner in CD8^+^ T cells from patient blood (44).

### PTPN2

PTPN2 has been recently classified as a robust biomarker for predicting prognosis and the efficacy of CIT. PTPN2 showed a positive correlation with tumor mutation burden in solid cancers. In addition, positive correlations with microsatellite instability were identified in lung adenocarcinoma (LUAD), stomach adenocarcinoma (STAD), colon adenocarcinoma (COAD), lung squamous cell carcinoma (LUSC), skin cutaneous melanoma (STES), HNSC, and breast cancer (BRCA). Negative correlations were discovered in DLBCL, glioblastoma and low-grade glioma (GBM/LGG) and pan-kidney cohort (KIPAN). PTPN2 was also positively correlated with neoantigens in COAD and BRCA (195).

The tumor immune dysfunction and exclusion (TIDE) algorithm was used to predict responses to cancer immunotherapy. Patients with high TIDE scores are more likely to be non-responsive to immunotherapy (196). Higher TIDE scores were observed in the PTPN2-high group, implying that increased PTPN2 expression in certain cancer types negatively impacted immunotherapy outcomes in patients (195). Importantly, increased PTPN2 expression was linked to poor prognosis in pancreatic adenocarcinoma (PAAD) (197). Overall, the evidence suggests that PTPN2 is a potential therapeutic target and diagnostic biomarker for specific cancers (195). In the ongoing clinical trial investigating the ABBV-CLS-484 PTPN1/2 inhibitor compound, microsatellite instability and PD-L1 expression are among the biomarkers used for patient selection (NCT04777994).

### SHP1

Numerous studies have investigated the prognostic and diagnostic value of Shp1 expression and promoter methylation to identify new cancer biomarkers. Decreased Shp1 expression and PTPN6 hypermethylation are linked to tumor staging, pathological differentiation, and poor survival in various cancers, including esophageal squamous cell carcinoma (ESCC) (198), endometrial carcinoma (EC) (199), high-grade glioma (HGG) (200), and NSCLC (201). In prostate cancer, methylation of the *PTPN6* promoter and decreased expression of Shp1 correlate with increased malignancy and poor prognosis (202). PTPN6 methylation in plasma, combined with clinical analysis, may serve as a promising biomarker for NSCLC diagnosis and prognosis. Indeed, a good correlation between cell-free circulating DNA methylation and matched tumoral tissue has been observed in patients with glioma and NSCLC (200, 203).

### SHP-2

Immunohistochemistry staining and mRNA expression of SHP-2 generally correlate well and can be used as a biomarker for response (204, 205).

SHP-2 has been shown to be overexpressed in clinical samples of NSCLC, and SHP-2 knockdown reduces the proliferation and migration of lung cancer cells. This suggests that co-inhibition of EGFR and SHP-2 is an effective approach to overcome EGFR^T790M^ mutation-acquired resistance to EGFR tyrosine kinase inhibitors, thus supporting SHP-2 expression as a new biomarker in NSCLC treatment (206).

In addition, it has been shown that expression of the EGF ligand EREG correlates with the response to the SHP-2 inhibitor SHP099 in HNSCC. This indicated that SHP099 blocks MEK/ERK and PI3K signaling when EREG levels are low, thus conferring sensitivity to the drug. On the other hand, high EREG levels and sustained MEK/ERK/PI3K signaling confers resistance to SHP-2 inhibition, thus representing a potential biomarker for patient selection (207).

Interestingly, the SHP-2 inhibitor BBP-398 is currently tested in combination with nivolumab in patients with KRAS-mutated tumors, with the GTPase KRAS status being used as a biomarker for patient stratification (NCT05375084, NCT05480865).

### ENPP1/TREX1

For the cGAS/STING pathway negative regulator ENPP1, treatment monitoring biomarker experience recently became available from the first-in-human clinical trial for the candidate RBS-2418 from Riboscience LLC (**Table 6A**). Compared to baseline serum samples, where cGAMP was undetectable, RBS2418 treatment led to fully stable cGAMP levels, in line with absence of ENPP1 enzyme activity. These results confirm that cGAMPs levels can be used as a biomarker for target engagement as the direct consequence of ENPP1 inhibition, preventing cGAMP degradation. In addition, this study also analyzed immune biomarkers predictive of anti-tumor responses. Increased peripheral cDCs, proliferation of CD4^+^ and CD8^+^ T cells, expansion of TCR clonotypes, and gene expression upregulation for granule proteins in cytotoxic T cells were reported after RBS-2418 treatment (169). The reliability of these biomarkers will be further informed by follow-up ORR data in this first-in-class clinical trial.

Interestingly, the downregulation of the cGAS/STING pathway itself was shown to be a predictive biomarker for certain cancer subtypes, opening up the potential to identify patient groups that could benefit from cGAS/STING re-enhancing immunotherapy agents such as ENPP1 or TREX1 inhibitors. As an example, in NSCLC elevated expression of cGAS/STING pathway components was observed in patients with localized disease compared to patients with metastatic disease. These observations translated into a superior ORR for the patient group expressing high levels of cGAS, STING, and TBK. Thus, these proteins can be considered predictive biomarkers for this patient subgroup (208).

### EP2/EP4

Similar to ENPP1, response-predictive clinical biomarker information for dual EP2/EP4 inhibitors is available from the first clinical trial for this drug class conducted by Tempest Therapeutics (**Table 5A**). Based on serum samples taken pre- and post-TPST-1495 treatment, reversed PGE2-mediated immunosuppression was monitored by the increase of tumor necrosis factor alpha (TNFα) expression levels. In addition, the urinary PGE2 metabolite (PGEM) was measured post-treatment as a biomarker (147). Additional biomarker data are available from clinical trials with EP4 inhibitors, which could be used for the ongoing studies with EP2/EP4 inhibitors. In a first-in-human phase 1 study of palupiprant, tumor-core biopsies taken before and after treatment were successfully analyzed for pharmacodynamic biomarkers, including immune cell infiltration (T cells, macrophages) and gene expression profile analysis (e.g. TNFα, CXCL10). In addition, blood samples were analyzed for immune-related circulating factors (e.g. cytokines and chemokines), which showed changes in EP4-regulated genes following palupiprant treatment, including downregulation of *PTGER4* (gene encoding the EP4 receptor) and upregulation of CD274 (gene encoding PD-L1). With regard to predictive biomarkers, patients with higher baseline tumor infiltration of T cells and type 2 macrophages were more likely to achieve stable disease upon palupiprant treatment rather than progressive disease (209). Intriguingly, scRNA-seq analysis indicates that the expression of EP2 (*PTGER2*) and EP4 (*PTGER4*) is negatively correlated with patient prognosis in different tumor types (LUSC, BRCA, LIHC, and OV). This underlines the conserved and critical function of the PGE2-EP2/EP4 axis in human cancer, and also supports the potential of EP2/EP4 expression analysis as a potential predictive biomarker for identification of patient populations that would especially benefit from EP2/EP4 receptor inhibition (210).

### A2aR, A2bR and CD73

Monotherapy trials with negative modulators of the adenosine pathway mostly showed modest ORR. Therefore, apart from combination regimen approaches, identification of reliable biomarkers to select patients that would benefit from negative modulation of the adenosine pathway is crucial. Overexpression of the relevant pathway components A2aR, A2bR, CD73, and CD39 in different solid tumors has been documented in several studies (211), including studies of CD73 in the TME, which was shown to serve as a prognostic biomarker for clinical outcomes (212). In line with this, overexpression of components such as A2aR and A2bR might be suitable for predicting a successful response of these tumor types to ADO pathway inhibitors (213). Further efforts have already been undertaken to identify the ideal set of biomarkers for characterization of patients that would benefit most from eADO pathway modulation. The “Adenosine Gene Signature” (AdenoSig) was established based on the expression of a gene set comprising *CXCL1*, *CXCL2*, *CXCL3*, *CXCL6*, *CXCL8*, *PTGS2*, and *IL1β*, with the aim to identify patients for A2aR antagonist treatment (214). In addition, the “Adenosine Signaling Score” was proposed (spanning the genes *PPARG*, *CYBB*, *COL3A1*, *FOXP3*, *LAG3*, *APP*, *CD81*, *GPI*, *PTGS2*, *CASP1*, *FOS*, *MAPK1*, *MAPK3*, *CREB1*) to correlate with A2aR signaling in human cancers and the response to immunotherapy (215).

## Conclusion

Encouraged by the clinical success of immune checkpoint blockade using therapeutic antibodies, an increased focus into intracellular immuno-regulatory proteins could be observed over the last years.

Here, we reviewed targets with negative immunomodulatory potential, which are currently at the center of drug discovery efforts for small molecule inhibitors. Two general concepts to induce or increase an anti-tumor immune response are pursued: the more targeted approach of directly blocking negative feedback loops in immune cells (T cells, NK cells, professional antigen-presenting cells) or tumor cells, and the more complex approach of preventing or reverting an immunosuppressive TME. Assessing which approach will result in better clinical efficacy is challenging because to date clinical efficacy is mostly predicted from preclinical *in vitro* and *in vivo* studies. The translatability of syngeneic mouse efficacy models to clinical outcomes is limited. The fact that most molecules show *in vivo* responses similar to mouse surrogates for the approved CPIs against PD-(L)1 is encouraging, but not a proof for single agent activity. In fact, most preclinical mouse models are characterized by a very high level of immune cell infiltration, making them very responsive to treatment. Nonetheless, continuous development of *in vivo* models will help generate more predictive preclinical data sets. This includes tumor models based on mice with an engrafted human adaptive immune system, chronic infection models causing T cell exhaustion, and syngeneic models with acquired CPI resistance, which will provide more comprehensive preclinical data with a better understanding of the mechanism of action of the respective molecule.

Many CPIs were first developed as single agents and thereafter tested in CPI combinations. So far, successful combinations include the regimen anti-PD1/anti-CTLA4 (nivolumab, ipilimumab) and anti-PD1/anti-LAG-3, respectively (nivolumab, relatilimab) (216, 217). Clinical development plans for small molecule immuno-modulators follow a similar strategy. Following a single arm dose-escalation group, most clinical trials include combination arms with CPIs. The goal is to either induce or boost the activity of CPI treatment or to break acquired CPI-resistance in CPI-experienced patients. Provided that clinical benefit is observed, it can be assumed that indications will be stepwise expanded to less immunogenic, and therefore less infiltrated, tumors. Initial data providing insight into whether small molecules indeed have single agent activity in CPI-resistant tumors are expected within the next two years.

## Author contributions

LS: Conceptualization, Visualization, Writing – original draft, Writing – review & editing. LG: Visualization, Writing – original draft. AR: Conceptualization, Writing – original draft, Writing – review & editing. FR: Conceptualization, Writing – original draft, Writing – review & editing.
